# Gut–brain circuits for fat preference

**DOI:** 10.1038/s41586-022-05266-z

**Published:** 2022-09-07

**Authors:** Mengtong Li, Hwei-Ee Tan, Zhengyuan Lu, Katherine S. Tsang, Ashley J. Chung, Charles S. Zuker

**Affiliations:** 1 Howard Hughes Medical Institute and Department of Biochemistry and Molecular Biophysics, Chevy Chase, MD USA; 2 Zuckerman Mind Brain and Behavior Institute, Columbia University, New York, NY USA; 3 Department of Biological Sciences, Columbia University, New York, NY USA; 4 Department of Neuroscience, Vagelos College of Physicians and Surgeons, Columbia University, New York, NY USA; 5 Present Address: Agency for Science, Technology and Research, Singapore, Singapore

**Keywords:** Neural circuits, Molecular neuroscience

## Abstract

The perception of fat evokes strong appetitive and consummatory responses^1^. Here we show that fat stimuli can induce behavioural attraction even in the absence of a functional taste system^2,3^. We demonstrate that fat acts after ingestion via the gut–brain axis to drive preference for fat. Using single-cell data, we identified the vagal neurons responding to intestinal delivery of fat, and showed that genetic silencing of this gut-to-brain circuit abolished the development of fat preference. Next, we compared the gut-to-brain pathways driving preference for fat versus sugar^4^, and uncovered two parallel systems, one functioning as a general sensor of essential nutrients, responding to intestinal stimulation with sugar, fat and amino acids, whereas the other is activated only by fat stimuli. Finally, we engineered mice lacking candidate receptors to detect the presence of intestinal fat, and validated their role as the mediators of gut-to-brain fat-evoked responses. Together, these findings reveal distinct cells and receptors that use the gut–brain axis as a fundamental conduit for the development of fat preference.

## Abstract (web summary)

Behavioural and genetic experiments in mice reveal gut-to-brain circuits driving the development of fat preference, one responding to intestinal sugar and fat using cholecystokinin signalling, and the other responding only to fat.

## Main

Populations in both developed and developing countries have experienced catastrophic increases in the consumption of processed foods high in sugar and fat^5^. These changes in dietary intake have been implicated in increased malnutrition, including over-nutrition linked to a wide range of metabolic disorders and related comorbidities^1,6,7^.

Sugar and fat are essential nutrients and, consequently, animals have evolved taste-signalling pathways that detect and respond to sweet and fat stimuli, leading to appetitive and consummatory behaviour^1,8^. Remarkably, mice that lack sweet taste receptors^8^ can still develop a strong behavioural preference for sugar^9^. This suggested the existence of a taste-independent signalling pathway driving sugar preference. Indeed, it was recently demonstrated that the development of sugar preference is mediated by the gut–brain axis, independently of the taste system^4^. Furthermore, artificial sweeteners, although capable of activating the same taste receptors as sugar on the tongue^8,10^, do not activate the gut–brain sugar circuit, and consequently do not create a preference^4^. Together, these findings revealed a gut-to-brain, post-ingestive intestinal sugar-sensing pathway driving craving and attraction to sugar^4,11–14^.

Here we focus our attention on the neural basis of fat preference. We demonstrate that fat, like sugar, uses the gut–brain axis to drive consumption. Then, we dissect the nature of the receptors and neuronal elements mediating the development of fat preference.

The discovery of post-ingestive mechanisms activated by foods rich in sugar and fat can provide valuable strategies to modulate our sugar- and fat-craving eating habits and help combat obesity and associated disorders, including diabetes and cardiovascular disease.

## The development of fat preference

**Fig. 1:**
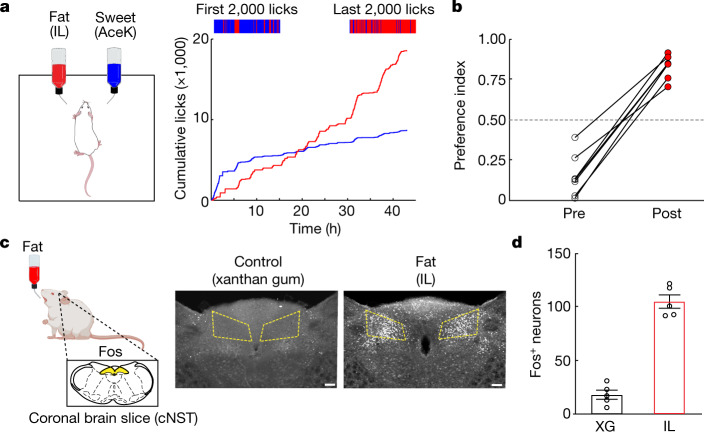
The development of fat preference. **a**, Left, cartoon illustrating the behavioural arena; mice were allowed to choose between a fat emulsion (1.5% Intralipid (IL)) and an artificial sweetener (3 mM AceK). Preference was tracked by electronic lick counters in each port. Right, cumulative licks for each bottle over the 48 h session. The colour bars at the top show lick rasters for fat (red) and sweet (blue) from the first and last 2,000 licks of the behavioural test. Note that by 24 h the mice begin to drink almost exclusively from the fat bottle (red trace). **b**, Preference plots for fat versus sweet. In these experiments, mice began the preference test preferring sweet (preference index < 0.5), but in all cases they switched their preference to fat (*n* = 7 mice, two-tailed paired *t*-test, *P* = 1.9 × 10^−5^) The dashed line indicates the equal preference level (50%). **c**, Schematic showing stimulation of Fos induction by fat ingestion. Strong Fos labelling is observed in the cNST (highlighted yellow) upon ingestion of 20% IL but not by the control stimulus (0.3% xanthan gum (XG)). Scale bars, 100 µm. **d**, Quantification of Fos-positive neurons. The equivalent area of the cNST (200 µm × 200 µm; bregma −7.5 mm) was processed, and positive neurons were counted for the different stimuli. Two-sided Mann–Whitney *U*-test between XG and IL (*n* = 5 mice), *P* = 7.9 × 10^−3^. Data are mean ± s.e.m.

**Extended Data Fig. 1:**
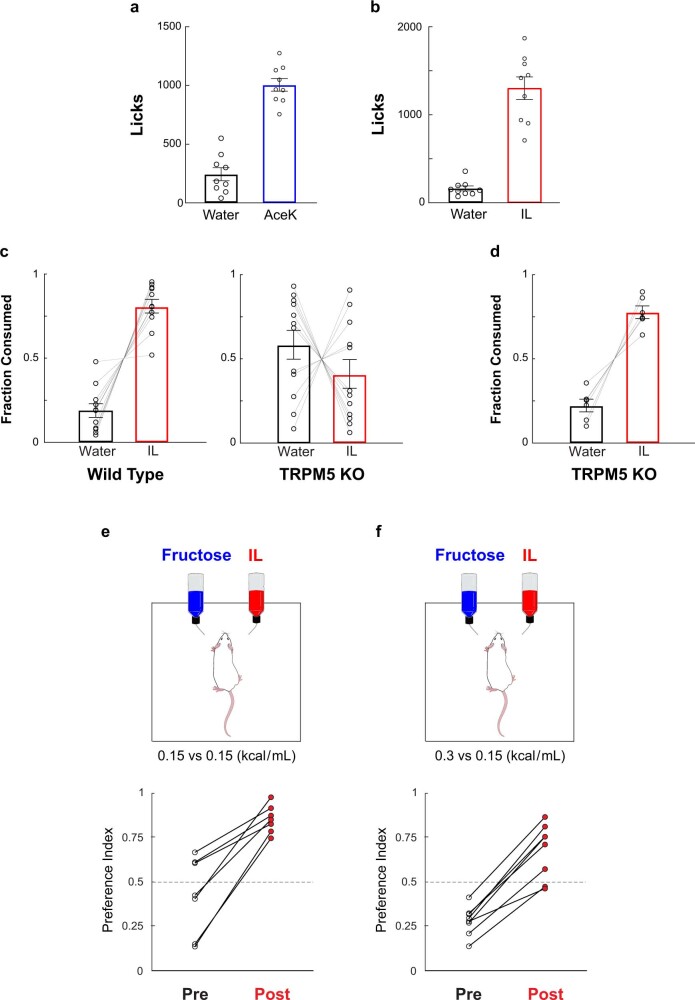
Development of post-ingestive fat preference is independent of immediate attraction to fat and caloric content. **a, b**, Immediate attraction to sweet and fat. Graphs of lick counts from brief-access (30 min) two-bottle tests. **a**, Artificial sweetener (3 mM AceK) versus water, *n* = 9 mice, two-tailed paired t-test, *P* = 2x10^−6^; **b**, Fat (1.5% Intralipid, IL) versus water, *n* = 9 mice, two-tailed paired t-test, *P* = 2x10^−5^. Values are mean ± s.e.m. Note strong innate attraction to sweet and fat stimuli. **c**, Immediate attraction to fat is abolished in TRPM5 knock-out animals. Shown are results from 30 min two-bottle test of fat (1.5% Intralipid, IL) versus water in wild type mice (left panel, *n* *=* 11 mice) versus TRPM5 knockout mice (right panel, *n* *=* 12 mice). TRPM5 knock-out animals are blind to the taste of fat^3^. Two-sided Mann–Whitney U-test wild type versus TRPM5 knockout fat consumption: *P* *=* 1.6x10^−3^. Note that there is no innate attraction to either bottle, with the animals randomly choosing to consume from either one. **d**, In contrast, in a 48 h two-bottle fat preference test, TRPM5 KO animals still developed strong post-ingestive preference to fat (*n* = 6 mice, two-tailed paired t-test, *P* = 7.4x10^−4^). **e**, Development of fat preference is independent of caloric content. To test the effect of calories, we examined preference between a caloric sugar (fructose) versus fat. Importantly, we used a sugar (fructose) that does not activate SGLT1, and therefore does not trigger post-ingestive preference^4^, thus we can isolate the effect of calories without the confound of having two preference-triggering stimuli (i.e. glucose versus fat). Cartoon on the top illustrates the behavioral arena; mice were allowed to choose between fructose (0.15 kcal/ml) and fat (IL at 0.15 kcal/ml). **f**, Similar test, but fructose at twice (0.3 kcal/ml) the caloric content of IL. By the end of the 48 h preference test, all the mice switched their preference for fat. **e**, paired t-test, *P* = 8x10^−4^, *n* = 7; **f**, paired t-test, *P* = 1x10^−5^, *n* = 7. Note that while at the higher fructose concentration (panel **f**) all of the animals began the test with much stronger attraction to the (sweeter) fructose bottle, all still switched their preference to fat, independent of caloric content.

To behaviourally monitor the development of post-ingestive fat preference, we presented mice with a choice between an artificial sweetener (3 mM acesulfame K (AceK)) and fat (1.5% Intralipid) (Fig. 1a). Both stimuli are innately attractive to a naive animal^8,15^ (Extended Data Fig. 1a,b), but artificial sweeteners do not trigger post-oral preference^4,16^. Therefore, this fat-versus-sweetener test enables us to monitor the emergence of fat preference from an initial state of no preference to a switch into a strongly appetitive stimuli. Indeed, our results showed that although mice initially preferred the artificial sweetener (Fig. 1a,b, pre), their preference is markedly altered within 24 h of exposure to both choices, such that by 48 h, the mice drink almost exclusively from the bottle containing fat (Fig. 1a,b, post). This behavioural switch illustrates the ability of fat stimuli to post-ingestively induce strong consummatory responses and appetitive behaviour^1^. This switch is also observed when comparing fat to an equicaloric sugar (Extended Data Fig. 1e,f), showing that calories are not driving the development of fat preference.

It was shown recently that the immediate attraction to fat is dependent on the TRPM5 channel expressed in taste receptor cells^3^ (Extended Data Fig. 1c). We hypothesized that if the development of fat preference is mediated via post-ingestive, rather than taste-evoked signalling, it should be independent of TRPM5 function, and consequently TRPM5-knockout mice should still be capable of developing behavioural preference for fat. As predicted, TRPM5-mutant mice, although blind to the taste of fat, remain fully capable of developing strong post-ingestive preference for fat^3^ (Extended Data Fig. 1d).

## Fat preference via the gut–brain axis

For an animal to develop a preference for fat over sweetener, it must distinguish between two innately attractive stimuli. We reasoned that if we could identify a population of brain neurons that respond selectively to the consumption of fat, it may provide an entry to reveal the neural control of fat preference and the basis for the insatiable appetite for fat.

**Extended Data Fig. 2:**
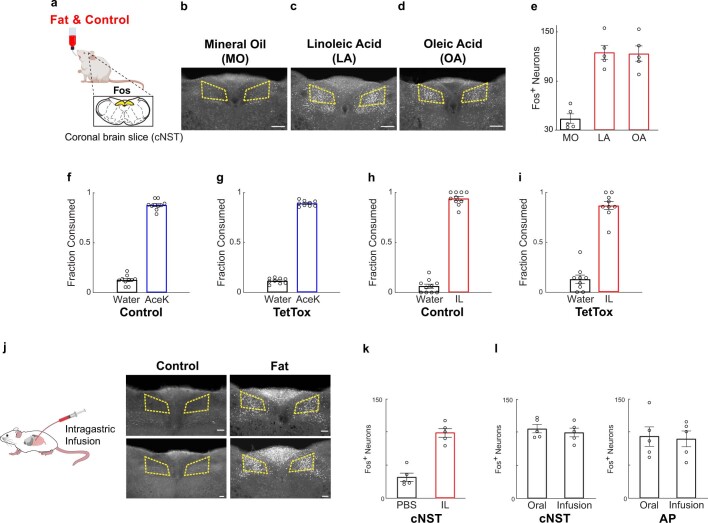
Fat activates cNST neurons. **a–d**, Strong Fos labelling is observed in neurons of the cNST (Bregma −7.5 mm) in response to ingestion of fat stimuli (panels **c-d**), but not in control animals (10% mineral oil, panel **b**). Stimulus: 10% linoleic acid (LA), 10% oleic acid (OA). Scale bars, 200 µm. **e**, Quantification of Fos-positive neurons. ANOVA with Tukey’s HSD test against mineral oil (MO, *n* = 5 mice): *P* = 3.4x10^−5^ for linoleic acid (LA, *n* = 5 mice), *P* = 3.9x10^−5^ for oleic acid (OA, *n* = 5 mice). Values are mean ± s.e.m. **f–i**, TetTox silencing of fat-TRAP cNST neurons does not impair immediate attraction to sweet (3 mM AceK; **f, g**) or fat (1.5% IL; **h, i**). Two-tailed paired t-tests: sweet versus water, wild type, *n* = 10, *P* = 1.1x10^−7^; TetTox *n* = 9, *P* = 1.1x10^−6^. For fat versus water, wild type, *n* = 10, *P* = 6.8x10^−5^; TetTox, *n* = 9, *P* = 1.7x10^−5^. Values are mean ± s.e.m. **j-k**, Intragastric infusion with fat activates cNST neurons. **j**, Direct intragastric infusion of fat (IL) but not control (PBS) robustly activates the cNST. Scale bars, 100 µm. **k**, Quantification of Fos-positive neurons in animals infused with control and IL stimuli, Two-sided Mann–Whitney U-test between control and Intralipid (*n* = 5 mice), *P* = 8x10^−3^. **l**, We note that we often observe variable labeling in the area postrema (see Fig. 1c and panel jhere), but such labeling is independent of oral versus intragastric infusion^4^. The bar graphs show the quantification of Fos^+^ neurons in the area postrema (AP) and cNST (Fig. 1d) in response to free licking of IL (90 min) versus intragastric infusion (*n* = 5 mice). cFos in cNST: oral 105 ± 6, infusion 99 ± 6, cFos in AP: oral 93 ± 15, infusion 90 ± 12. The equivalent area of the cNST (bregma − 7.5 mm) was processed and counted for the different experiments. Two tailed unpaired t-test, cNST: *P* = 0.54; AP: *P* = 0.86. Values are mean ± s.e.m.

We exposed separate cohorts of mice to three different lipid stimuli (Intralipid, linoleic acid or oleic acid) and to fat-free textural controls (xanthan gum or mineral oil). Using Fos as a proxy for neural activity^4,17^, we found that fat, but not control stimuli, elicited strong bilateral activation of neurons in the caudal nucleus of the solitary tract (cNST) in the brainstem (Fig. 1c,d and Extended Data Fig. 2a–e). The cNST is a nexus of interoceptive signals conveying information from the body to the brain via the gut–brain axis^18,19^. If the fat-activated brain cNST neurons are receiving signals originating in the gut, then direct delivery of fat stimuli into the gut should also induce activation of the cNST. We implanted an intragastric catheter in the stomach^4^ and infused either a fat solution or a vehicle control. As predicted, intragastric infusion of fat, but not of a vehicle, was sufficient to activate the cNST (Extended Data Fig. 2j–l).

**Fig. 2:**
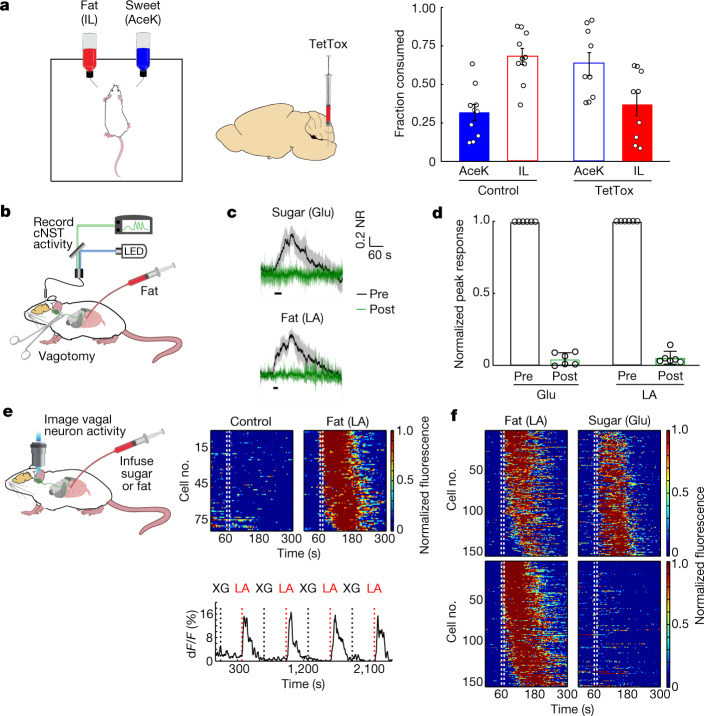
Fat preference is mediated by the gut–brain axis. **a**, Left, schematic for silencing fat-stimulated cNST neurons. A TetTox virus was targeted bilaterally to the cNST of TRAP2 mice for silencing. Right, the fraction of AceK versus IL consumption after the 48 h preference test, in control (*n* = 10) versus TetTox mice *(n* = 9). Two-sided Mann–Whitney *U*-test for fat, *P* = 1.4 × 10^−3^ (total volume consumed: control, 9.9 ± 2.3 ml; TetTox, 8.3 ± 2.1 ml). Control mice developed a strong preference for IL versus sweetener. By contrast, mice in which fat-activated cNST neurons have been silenced do not show a preference for fat over sweetener. Data are mean ± s.e.m. **b**, Fibre photometry was used to monitor activity in cNST neurons in response to intestinal delivery of fat. **c**, Neural responses following 10 s intestinal delivery of fat (10% linoleic acid (LA)) or control sugar (500 mM glucose (Glu)). The solid trace is the mean and the shaded area represents s.e.m. Responses after bilateral vagotomy are shown in green. Note total loss of responses following bilateral vagotomy^4^. *n* = 6 mice. NR, normalized response. **d**, Quantification of neural responses pre- and post-vagotomy. Two-tailed paired *t*-test, *P* = 4.6 × 10^−8^ (sugar), *P* = 4.9 × 10^−8^ (fat). Data are mean ± s.e.m. **e**, Imaging of calcium responses in vagal neurons as stimuli are delivered to the intestines. Heat maps depict *z*-score-normalized fluorescence traces from vagal neurons identified as fat responders (*n* = 84 out of 515 cells from 8 ganglia). Each row represents the average activity of a single cell to four trials. Stimulus window (10 s) is indicated by dotted white lines. Note the strong responses to intestinal delivery of fat (10% LA) but not to control stimuli (0.1% XG plus 0.05% Tween 80). Shown below are sample traces of responses to alternating 10 s pulses of control (XG) and fat stimuli (LA). **f**, Heat maps depict z-score-normalized responses to interleaved 10 s stimuli of fat (10% LA) and sugar (500 mM Glu). Each row represents the average activity of a different neuron during three exposures to the stimulus. Top, 151 neurons that responded to intestinal application of both fat and glucose. Bottom, a separate pool of 153 neurons that responded only to fat. *n* = 22 vagal ganglia; 1,813 neurons were imaged.

**Extended Data Fig. 3:**
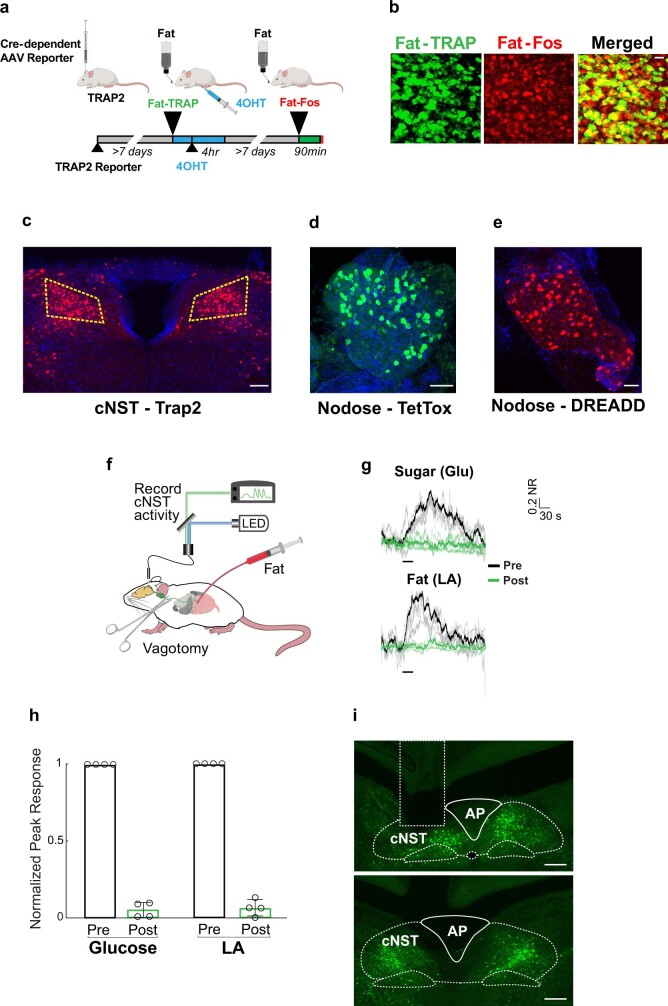
Quantification of cNST and nodose labeling. **a**, Genetic TRAPing of cNST neurons with fat stimuli (see Methods) is efficient and reliable. Note that animals must not be food deprived to prevent labeling unrelated circuits (Methods). We labelled the fat-induced TRAP2 neurons by infection with an AAV carrying a Cre-dependent fluorescent reporter^4^ (shown in green), and then performed a second cycle of fat stimulation followed by Fos antibody labelling^4^ (shown in red; see Methods). **b**, By comparing the number of neurons expressing the fluorescent reporter to the number neurons labelled by Fos antibodies, we determined that 90.7 ± 0.6% of Fat-Fos neurons were also TRAPed with the fat stimuli (*n* = 6). Scale bar, 20 μm. **c**, For experiments targeting AAV- FLEX-TetTox, or AAV-DIO-mCherry (or GFP) to the cNST we used fat-stimulated TRAP2 animals (see Methods). By comparing the number of neurons expressing AAV after TRAPing and infection, to the number of cNST neurons labeled after crossing similarly TRAped animals to Ai9^63^ reporter mice, we estimate the infection of TRAPed neurons to be >90%: TRAP-AAV: 68 ± 1 neurons; Trap-Ai9: 71 ± 1 neurons (*n* = 8). Scale bar, 100 μm. The equivalent area of the cNST (bregma − 7.5 mm) was processed and counted for the separate experiments. Values are mean ± s.e.m. **d**, Shown is a whole mount image of a nodose ganglia from *Vip-Cre* animals infected with AAV- FLEX-TetTox virus (see Methods). Average number of labeled neurons from Vip-TetTox was 48 ± 13 neurons (*n* = 4), and the average of nodose neurons labeled with AAV- FLEX-TetTox virus in the *Trpa1-Cre* animals was 62 ± 23 neurons (n = 6; not shown). These numbers compare favorably (~50%) to the total number of VIP and Trpa1 neurons detected by crossing the Cre animals to reporter Ai9^63^ mice: VIP ~100 neurons; Trpa1 ~120 neurons (data not shown). Scale bar, 100 μm. **e**, Shown is a whole mount image of nodose ganglia from *Vip-Cre* animals infected with AAV- DIO-hM3Dq (activator DREADD^36,38^). VIP-DREADD labeling efficiency: 43 ± 4% (43 ± 4/100.5), *n* = 9. Scale bar, 100 μm. **f-i**, cNST-activation in response to intestinal delivery of fat and sugar is mediated via vagal signaling. AAV carrying a Cre-dependent GCaMP6s was targeted to the cNST of *Penk-Cre* animals^4^. **f**, Fiber photometry was used to monitor cNST activity in response to intestinal delivery of sugar and fat stimuli (see also Fig. 2b–d); to minimize any labeling in the AP and ensure the signals originate in cNST neurons, we used AAV targeting of GCaMP6s to the cNST (see panel I below). **g**, Neural responses following intestinal delivery of fat (10% linoleic acid, LA) or sugar (500 mM glucose, Glu). The light traces denote normalized three-trial averages from individual animals, and the dark trace is the average of all trials. The responses after bilateral vagotomy are shown in green. Black bars below traces indicate the time and duration of stimuli; *n* = 4 mice. NR, normalized response. Note robust, time-locked responses of cNST neurons to intestinal delivery of fat and sugar. Importantly, responses are abolished after bilateral vagotomy. **h**, Quantification of neural responses before and after vagotomy. Two-tailed paired t-test, P = 3.8x10^−5^ (sugar), *P* = 5x10^−5^ (fat). Data are mean ± s.e.m. **i**, Sample brains of two different injected animals demonstrating expression of GCaMP6s restricted to the cNST, with minimal expression in the AP; the top brain also demarks the location of the recording fiber (dashed rectangle). Scale bars, 200 μm.

Next, we reasoned that if the fat-activated cNST neurons are essential for creating fat preference, then blocking their function should prevent the development of fat preference. We used the targeted recombination in active populations (TRAP) system^20^ to target Cre recombinase to fat-activated cNST neurons, and bilaterally injected an adeno-associated virus (AAV) carrying a Cre-dependent tetanus toxin light chain^21^ (TetTox) construct to genetically silence synaptic transmission in the cNST neurons responding to fat (Fig. 2a and Extended Data Fig. 3).

To ensure that the genetic silencing did not affect the immediate attraction to fat (that is, the taste-dependent innate attraction), they were first tested in a standard fat-versus-water two-bottle discrimination assay. Our results showed that the silenced mice still exhibited normal immediate attraction to fat, and were indistinguishable from controls (Extended Data Fig. 2f–i). By contrast, they were unable to develop post-ingestive preference for fat, even after prolonged testing sessions (Fig. 2a).

## Fat and sugar activated vagal neurons

To investigate how fat signals are transferred from the gut to the brain, we infused fat stimuli into the gut, and used fibre photometry to simultaneously record neural activity in cNST neurons^4^ (Fig. 2b). Our results showed that cNST neurons are robustly activated by direct intestinal infusion of fat, with responses tracking the delivery of the stimulus (Fig. 2c,d and Extended Data Fig. 3f–i).

The vagus nerve serves as a key conduit for conveying information from the gut to the brain^4,12,13,19,22^. If the vagus nerve is required for the transmission of fat signals from the gut to the cNST, then transection of the vagus nerve should prevent the signals from reaching the brain. To test this, we infused the gut with fat (or sugar as a control^4^) and recorded stimulus-evoked responses in the cNST. Indeed, fat-activated neural responses in the cNST were effectively abolished after bilateral vagotomy (Fig. 2c,d and Extended Data Fig. 3f–i), thus establishing the vagus nerve as the conduit for transmitting the fat signal from the gut to the brain.

**Extended Data Fig. 4:**
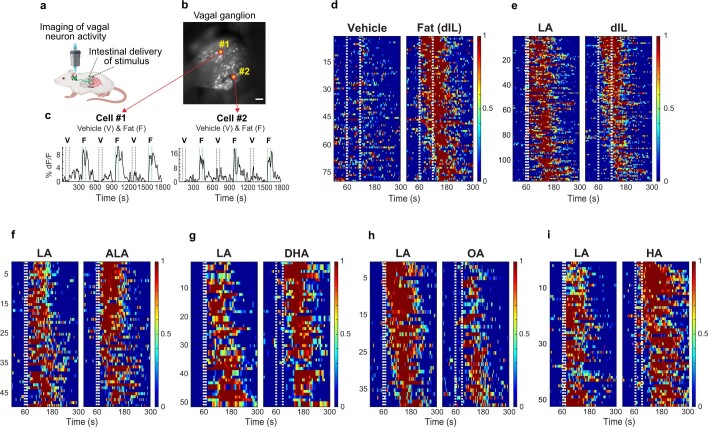
Various dietary fatty acids activate vagal neurons. **a-b**, Schematic of vagal calcium imaging while simultaneously delivering stimuli into the intestines (see Methods for details). The picture shows a representative view of a vagal nodose ganglion of *Vglut-Cre; Ai96* in an imaging session. Two fat responders (denoted #1 and #2) are highlighted, and their responses shown in panel c. **c**, Sample traces of vagal responses to intestinal stimulation with alternating pulses of vehicle or fat (pre-digested IL; see Methods for details). Note time-locked, reliable responses to fat, but not to vehicle control. Stimulus window (60 s) is marked by dotted white lines. Note that since IL is a complex mix, it must be pre-digested in vitro by incubation with lipases prior to using in imaging experiments (versus ingestion, where endogenous lipases in the stomach naturally digest IL). **d**, Heat maps depict z-score-normalized fluorescence traces from vagal neurons that responded to pre-digested (dIL, *n* = 79/463 neurons from 8 ganglia). Each row represents the average activity of a single cell to three trials. Stimulus window is shown by dotted white lines. **e–i**, Responses of vagal neurons to intestinal delivery of a range of fatty acids. **e**, Heat maps show z-score-normalized fluorescence traces of vagal neurons to intestinal delivery of 10% LA (10 s) and digested Intralipid (dIL); *n* = 116/634 neurons from 7 ganglia; note that the same neurons responded to both stimuli. **f**, 10% LA (10 s) and 10% alpha-linolenic acid (ALA), *n* = 49/322 neurons from 3 ganglia; **g**, 10% LA (10 s) and 10% docosahexaenoic acid (DHA), *n* = 51/348 neurons from 5 ganglia; **h**, 10% LA (10 s) and 10% oleic acid (OA), *n* = 39/418 neurons from 6 ganglia; **i**, 10% LA (10 s) and 10% hexanoic acid (HA), *n* = 52/495 neurons from 6 ganglia.

To directly examine and monitor the fat responses of vagal sensory neurons, we carried out functional imaging of the nodose ganglion (which contains the cell bodies of vagal neurons). We targeted the genetically encoded calcium indicator GCaMP6s^23^ to vagal sensory neurons using *Vglut2-cre* mice^4,24,25^ (*Vglut2* is also known as *Slc17a6*), and used a one-photon calcium imaging setup coupled to synchronous intestinal delivery of fat to record neuronal responses in vivo^4^ with real-time kinetics (Fig. 2e and Extended Data Fig. 4a–c). To administer the stimuli, a catheter was placed into the duodenal bulb, and an exit port was created by transecting the intestine 10 cm distally. During each imaging session, the intestine was exposed to a pre-stimulus application of PBS, a 10 s (33 μl) exposure to the fat or sugar stimuli (limited to 10 s to prevent activation of non-selective osmolarity responses^4,24^), and a 180 s post-stimulus wash (see Methods for details); this regime was repeated at least 3 times for each stimulus. Using this preparation, we showed that intestinal infusion of fat (for example, linoleic acid), but not vehicle control, evoked robust responses in a unique subset of vagal neurons (Fig. 2e and Extended Data Fig. 4c,d); the responses were reproducible and time-locked to stimulus delivery (Fig. 2e and Extended Data Fig. 4c). These neurons responded to a variety of dietary fatty acids (Extended Data Fig. 4d–i), thus defining a distinct class of vagal neurons that are reliably activated by intestinal fat stimuli.

We showed previously that intestinal application of glucose also activates a subset of vagal neurons^4^, and demonstrated that these, in turn, are part of the essential gut–brain axis driving the development of sugar preference. Next, we sought to examine how vagal neurons respond to these fat and sugar nutrient signals in the gut.

**Fig. 3:**
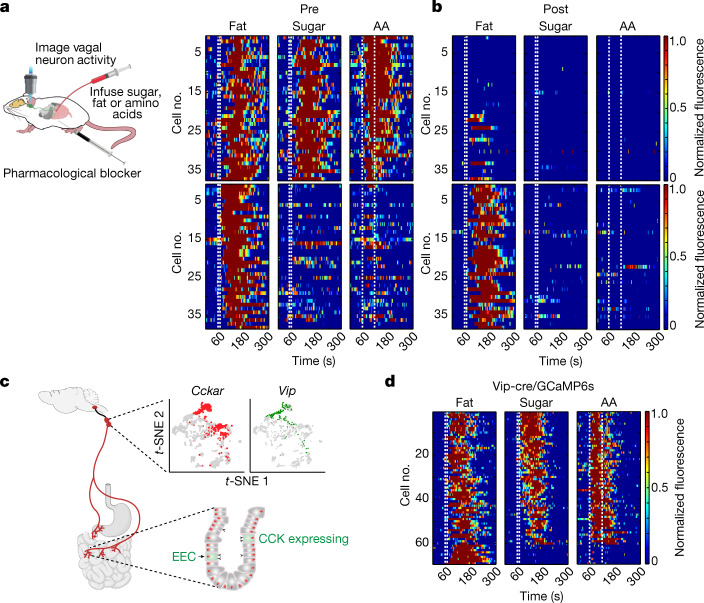
Nutrients engage gut-to-vagal CCK-mediated signalling. **a**, Imaging of calcium responses in vagal sensory neurons^4^ while delivering fat (10% LA), sugar (500 mM Glu) or amino acid (250 mM amino acid mixture) (AA) stimuli to the intestines (Methods). Heat maps depict *z*-score-normalized fluorescence traces of sugar/nutrient responders (top) and fat-only responders (bottom) from 641 neurons of 8 mice, before application of CCKAR blocker (pre). The stimulus window (10 s for fat or sugar, 60 s for amino acids) is indicated by dashed white lines. **b**, To inhibit CCK signalling, we applied devazepide^11^ (4 mg kg^−1^, 200 μl), a CCKAR antagonist^28^ (post) (Methods). Top, note that blocking CCKAR receptor activation abolishes sugar-, fat- and amino acid-evoked activity in nearly all the nutrient responders (compare with **a**, top). Bottom, by contrast, the CCKAR blocker had no effect on the fat-evoked activity in the fat-only responders (compare with **a**, bottom). See Extended Data Fig. 6 for results using glutamate receptor blockers. **c**, Cartoon of the gut-to-brain sugar/nutrient-sensing vagal axis. Bottom right, an expanded view of CCK-expressing EECs in the intestines. Top right, two-dimensional *t*-distributed stochastic neighbour embedding (*t*-SNE) plot of the transcriptome of mouse vagal nodose neurons^37^. Clusters expressing *Cckar* are shown in red and clusters expressing *Vip* are shown in green (Methods). **d**, Calcium responses in vagal ganglia of mice expressing GCaMP6s in VIP neurons during infusion of fat, sugar or amino acids stimuli into the intestines. Heat maps show *z*-score-normalized fluorescence traces. Approximately 30% of VIP vagal neurons responded to nutrient stimuli (*n* = 60 out of 203 neurons from 9 ganglia), but only a small fraction (~4%) responded to fat. Stimuli: 10% LA, 500 mM Glu or 250 mM amino acid mixture.

**Extended Data Fig. 5:**
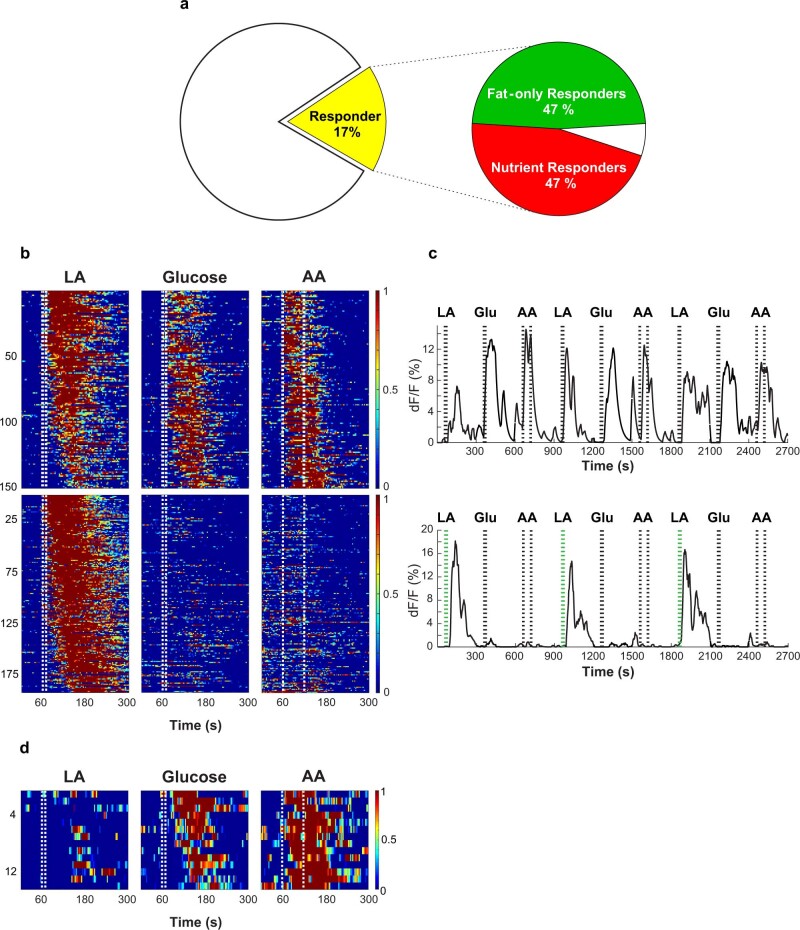
Distinct populations of vagal neurons respond to intestinal delivery of nutrients and fat. **a**, Pie chart illustrating the fraction of fat and sugar responders in the nodose ganglia of Vglut2-GCaMP6s animals. The data is from 1813 neurons from 22 ganglia (red, *n* = 323 cells, 17%). Right, within the responding neurons, 151 (47%) responded to both sugar and fat (“nutrient responders”), while 153 (47%) responded only to fat but not to sugar stimuli (“fat-only responders”). **b**, Sugar/nutrient versus fat-only vagal neurons: Heat maps depicting z-score-normalized vagal responses to intestinal delivery of fat (10% linoleic acid, LA), sugar (500 mM glucose) and amino acids (250 mM amino acid mixture, AA; see Methods). Each row represents the average activity of a single cell to 3 trials. Stimulus window is shown by dotted white lines. Upper panels show 150 neurons that responded to intestinal application of sugar, fat and amino acids (“sugar/nutrient responders”); bottom panels show 192 neurons that responded only to fat. *n* = 22 ganglia, 1884 imaged neurons. **c**, Representative traces from a “sugar/nutrient responder” (top) and a “fat-only responder” (bottom). Shown are responses to intestinal stimulation with 9 interleaved pulses of fat (10% LA, 10 s, green dotted line), sugar (500 mM Glu, 10 s) and amino acids (250 mM AA, 60 s). **d**, Heat maps of the small subset of vagal neurons that responded to sugar and amino acids but not to fat (*n* = 14/1884 neurons from 22 nodose ganglia). On average, less than 1 neuron was detected per ganglia. We note that when using high concentrations of glucose stimuli (>250 mM) for long stimulation times (60 s) one can detect strong vagal responses, but these have been shown not to be sugar-preference responses^4,22^.

We recorded the activity of vagal neurons to alternating gut stimulation with fat and sugar (10 s of 10% linoleic acid and 10 s of 500 mM glucose). Out of more than 1,800 vagal sensory neurons examined from 22 nodoses, we identified two distinct groups of vagal neurons. One group (around 8% of the total imaged neurons) responded to both sugar and fat. The other, a non-overlapping group (also around 8% of the neurons), responded only to fat, but not to sugar (Fig. 2f and Extended Data Fig. 5a). Notably, the subset responding to sugar and fat was also activated by amino acids (Fig. 3a and Extended Data Fig. 5b,c). These results defined two distinct populations of vagal neurons: one, hereafter referred to as sugar/fat responders, function as sensors for all three essential macronutrients in the gut: sugar, proteins and fat. The other population, hereafter referred to as fat-only responders, responds selectively to intestinal delivery of fat. We note that less than one neuron per nodose was found to respond to intestinal delivery of sugar or amino acids but not fat (Extended Data Fig. 5d); however, given such small numbers, these were not considered further (it is likely that they represent sugar or nutrient responders with very small responses to fat).

## Fat and sugar signalling in the gut

**Extended Data Fig. 6:**
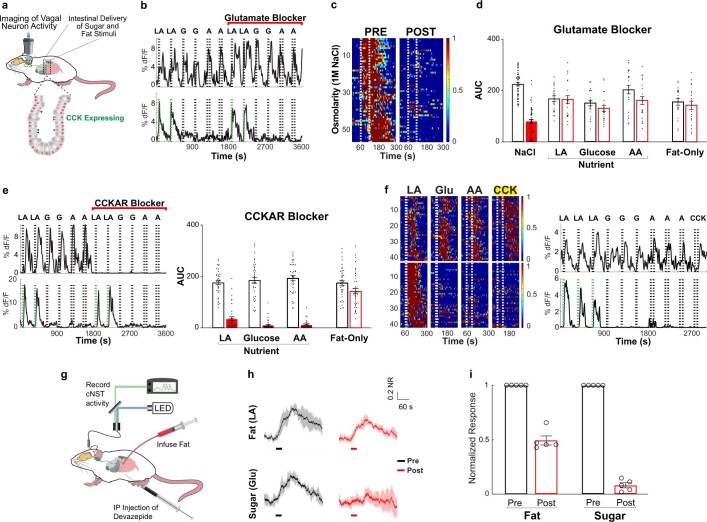
CCK signalling not glutamate mediates sugar/nutrient responses. **a**, Cartoon of vagal calcium imaging while simultaneously delivering sugar and fat stimuli into the intestines. The bottom inset illustrates CCK-expressing enteroendocrine cells (EECs) in the intestines. **b**, A recent study^11^ reported that the gut-to-vagal sugar preference signal is carried by glutamate as a transmitter^26^. However, this conclusion was based on three indirect assays and measurements. First, the use of in vitro organoids with dissociated vagal neurons, where all native connectivity between potential EECs and vagal neurons is lost^26^. Second, by using whole nerve recordings from thousands of random vagal fibres^11^, which do not afford the identification of the functionally relevant vagal signal (i.e. recognizing the sugar-preference signals from any other activity). Third, by using very long sugar stimuli (1 min) under conditions known to activate large populations of vagal neurons that mask the response of sugar/nutrient preference neurons^4,22,58^ (note also that the whole vagal nerve responses, unlike sugar/nutrient responses, never decayed after termination of the sugar stimulus). Consequently, we directly examined the role of glutamate signalling by imaging the responses of the relevant sugar-preference vagal neurons to intestinal sugar stimuli before and after addition of a mixture of AP3 and KA glutamate receptor antagonists. Indeed, our results demonstrated that pharmacological inhibition of glutamate-based signalling has no effect on this gut-to-vagal sugar/nutrient sensing circuit. Shown are representative traces of vagal neuron responses to intestinal infusions of fat, sugar and amino acids before and after treatment with ionotropic/metabotropic glutamate receptor antagonists (2 mg/kg AP3 with 300 μg/kg kynurenic acid, see Methods). Top traces show sugar/fat/amino acid responding vagal neurons, bottom traces show fat-only responders. **c**, In contrast, pharmacological inhibition of glutamate-based signalling abolished all osmolarity responses. Heat maps depicting z-score-normalized vagal responses to intestinal osmolarity stimuli (60 s of 1 M NaCl)^4,22,58^ before and after treatment with ionotropic/metabotropic glutamate receptor antagonists (2 mg/kg AP3 with 300 μg/kg kynurenic acid). **d**, Quantification of the responses to 1 M NaCl, 10% LA, 500 mM Glucose, and 250 mM AA mixture before (black bars) and after blockers (red bars). 1 M NaCl, *n* = 56 neurons, *P* = 1x10^−10^. For nutrient responders: LA, *n* = 21, *P* = 0.16; Glucose, *n* = 21, *P* = 0.85; AA, *n* = 21, *P* = 0.07. For fat-only responders, *n* = 19, *P* = 0.54 by two-tailed paired t-tests. All values are mean ± s.e.m. AUC: average area under curve (see Methods). **e**, Left, Representative traces of vagal neuron responses to intestinal infusions of fat, sugar and amino acids before and after treatment with cholecystokinin A receptor (CCKAR) blocker (4 mg/kg devazepide, see Methods). Note robust, reliable responses to fat (10% LA) and sugar (500 mM Glucose) prior to addition of CCKAR antagonist. However, all responses are loss after addition of antagonist (top panel). By contrast, fat-only responses are unaffected (bottom panel). Right panel, quantification of responses before (open bars) and after (red bars) CCKAR antagonist (data from Fig. 3a, b). For nutrient responders: LA, *n* = 37 neurons, *P* = 1x10^−9^; Glucose, *n* = 37, *P* = 1x10^−9^; AA, *n* = 37, *P* = 1x10^−9^. For fat-only responders, *n* = 38, *P* = 0.11 by two-tailed paired t-tests. All values are mean ± s.e.m. **f**, Sugar/nutrient but not fat-only responders utilise CCK signalling. Left, Heat maps depicting z-score-normalized fluorescence traces from vagal neurons identified as sugar/nutrient responders (upper panels, *n* = 41 neurons); note responses to sugar, fat and amino acid stimuli. The lower heat-map shows the fat-only neurons (*n* = 41 neurons). After stimulating with CCK (1 μg/ml), all sugar/nutrient responders were activated, but not the fat-only vagal neurons. Right, Representative traces of 2 sample neurons to pulses of 10% linoleic acid (LA), 500 mM glucose (G), 250 mM amino acids (A), and CCK. Stimulus windows are indicated by dotted lines. **g-h**, CCK-dependent (sugar and fat) and CCK-independent (fat-only) intestinal gut-to-brain fat-preference pathways co-contribute to fat signals in the cNST. Shown are photometric recordings of cNST neurons in *Penk-Cre* animals^4^ in response to intestinal fat-evoked activation of both fat-stimulated vagal pathways (black traces and bars). Shown in red are the same responses after inhibiting signaling via the CCK-dependent vagal pathway (see panel a- f). **i**, cNST responses to intestinal fat stimulation are reduced to ~50% after removing CCK-dependent signaling, demonstrating the separate contributions of the two fat-preference circuits. As expected, sugar-evoked responses are completely abolished after inhibiting signaling via the CCK-dependent pathway. *n* = 5, *P* = 2.4x10^−6^ by two-tailed paired t-test. All values are mean ± s.e.m. See text and methods for details. We note that in gain-of-function experiments, with DREADD being overexpressed in vagal neurons, activation of a single pathway is sufficient to create new preferences (see for example Fig. 4).

We next explored how sugar or nutrient signals are transmitted from the gut to vagal neurons. Cholecystokinin (CCK)-expressing enteroendocrine cells (EECs) in the intestine have been proposed to function as the sugar-preference gut sensing cells^11,26^. We hypothesized that CCK may be the signal between the gut and their partner vagal neurons. We thus examined responses of vagal neurons to intestinal application of sugar, fat and amino acids, before and after pharmacologically inhibiting CCK signalling with devazepide^27^, a CCK-A receptor^28^ (CCKAR) antagonist (Fig. 3a). Indeed, blocking CCK signalling abolished all the responses of the vagal sugar/fat neurons (that is, to intestinal stimulation with sugar, fat and amino acids). By contrast, the fat-only responses remained robust and reliable (Fig. 3a,b and Extended Data Fig. 6). Given these results, we anticipated that the application of CCK should strongly activate the nutrient responding vagal neurons, but not the fat-only neurons. Our results showed both predictions to be correct (Extended Data Fig. 6f). Finally, we also examined the potential role of glutamate signalling^11^ by imaging responses of vagal neurons to intestinal sugar stimuli before and after addition of a mixture of l-(+)-2-amino-3-phosphonopropionic acid (AP3) and kynurenic acid, two glutamate receptor antagonists^29,30^. Our results demonstrated that pharmacological inhibition of glutamate-based signalling has no effect on the gut-to-vagal sugar/nutrient-sensing circuit (Extended Data Fig. 6a–d). Together, these results substantiate CCK as the transmitter mediating sugar/nutrient-sensing in the gut–brain axis, and further distinguishes the CCK-dependent from the CCK-independent fat-sensing gut-to-brain pathways.

## Nutrient responders in the nodose

Given that gut sugar, fat and amino acid responders rely on CCK signalling, we expected that vagal neurons receiving this gut-to-brain signal would be defined by the expression of CCK receptors (such as CCKAR) (Fig. 3c). CCK is principally known as a satiety hormone, whose role is to modulate food intake by suppressing appetite^31,32^. By contrast, the function of nutrient preference circuits is to promote nutrient consumption^1,4^. Thus, we explored how CCK can function both as a satiety hormone and as a nutrient preference signal in the gut. We reasoned that this conundrum could be easily resolved if a genetically distinct^33^ subset of CCKAR-expressing vagal neurons mediates nutrient preference.

**Extended Data Fig. 7:**
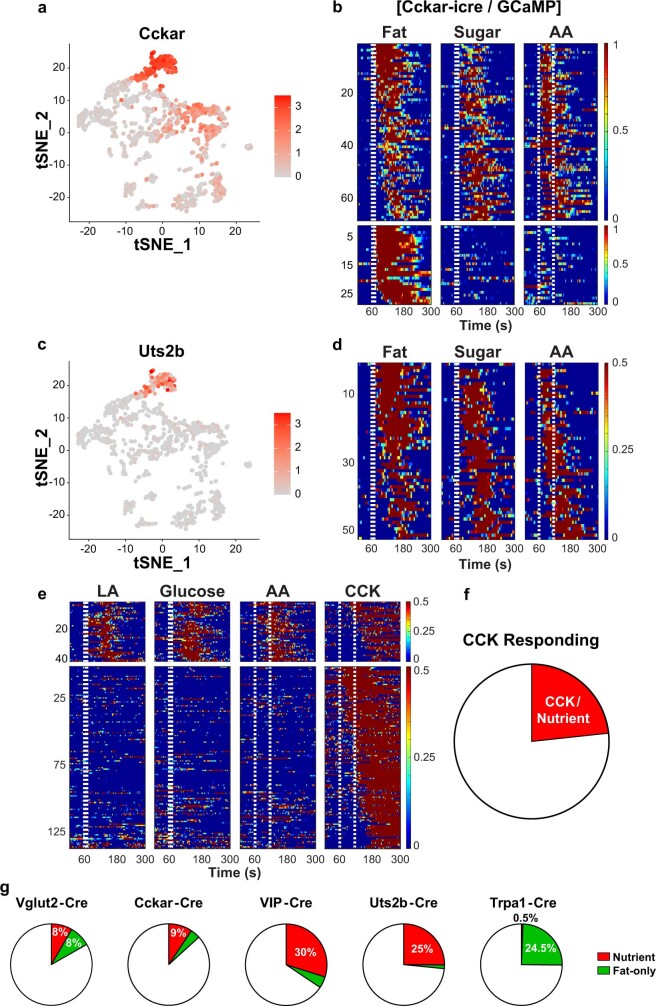
Sugar/fat/amino acid sensing vagal neurons. **a**, Shown is a tSNE plot of the transcriptome of mouse vagal nodose neurons (original data set taken from reference^37^); CCKAR expression is represented on a grey-to-red scale. **b**, CCKAR-expressing neurons respond to intestinal stimulation with nutrients. An engineered *Cckar-iCre* was used to drive GCaMP6s expression in CCKAR vagal neurons (see Methods). We analysed 724 imaged neurons from 12 ganglia. Shown are heat maps depicting z-score-normalized fluorescence traces of the CCKAR-expressing neurons responding to intestinal delivery of fat (10 % linoleic acid), sugar (500 mM glucose) or amino acids (250 mM amino acid mixture). Stimulus window is shown by dotted white lines. **c-d**, tSNE plot of the transcriptome of mouse vagal nodose neurons; urotensin 2B (*Uts2b*) expression is represented on a grey-to-red scale. **d**, Shown are responses of vagal Uts2b-expressing neurons (Uts2b-GCaMP6s) to intestinal delivery of fat (10 % linoleic acid), sugar (500 mM glucose) or amino acids (250 mM amino acid mixture). The heat maps depict z-score-normalised fluorescence traces of sugar/nutrient responders (*n* = 52/207 neurons from 7 ganglia). Stimulus window is shown by dotted white lines. Note that only 3 of the 52 neurons responded only to fat (shown at the top of the heat maps). **e**, Sugar/nutrient responders are a unique subset of CCKAR-expressing vagal neurons. Heat maps showing z-score-normalized fluorescence traces from vagal neurons that respond to CCK and nutrient stimuli (see Extended Data Fig. 6f). While all of the neurons that responded to intestinal stimulation with sugar, fat and amino acids (i.e. the sugar/nutrient sensors) also responded to CCK, the vast majority of vagal neurons that respond to CCK do not respond to nutrient stimuli (bottom heat maps, *n* = 136 neurons). This is expected since only a small fraction would be mediating sugar/nutrient preference, versus other roles of CCK signaling^31–33^. Stimuli: 10% linoleic acid (LA), 10 s; 500 mM glucose, 10 s; 250 mM amino acids (AA), 60 s; 1 μg/ml CCK, 60 s. **f**, the pie chart is based on data from 12 ganglia. Since vagal neurons that only respond to fat stimuli are not activated by CCK, they are not part of this analysis (see Extended Data Fig. 6f, bottom panels). **g**, Pie charts depicting the fraction of sugar/nutrient (red) and fat-only (green) responders in animals driving GCaMP6s reporter from various driver lines: *Vglut2-Cre, Cckar-Cre, Vip-Cre, Uts2b-Cre*, and *Trpa1-Cre* animals. *VIP/Uts2b* define the sugar/nutrient responders while TrpA1 mark the fat-only responders.

We engineered *Cckar-cre* mice by targeting Cre recombinase to the *Cckar* gene^34^ (Methods), and used them to functionally validate the nutrient-evoked activation of CCKAR vagal neurons (Extended Data Fig. 7a,b). Next, we used single-cell RNA-sequencing (RNA-seq) data from the nodose ganglion^35–37^ to further characterize subsets of CCKAR-expressing neurons, and generated Cre driver lines expressing GCaMP6s in subsets of candidate clusters. Our results showed that a unique pool of CCKAR-expressing vagal neurons marked by expression of the vasoactive intestinal peptide (VIP) labelled the nutrient responders (with only a small fraction of the fat-only neurons) (Fig. 3c, d, Extended Data Fig. 7e–g). We then further refined this cluster by removing the small number of fat-only responding neurons (Extended Data Fig. 7c,d,g). These results validate the segregation of the nutrient versus the fat-only circuit, and substantiate CCK in the gut as the transmitter mediating sugar/nutrient signals.

**Fig. 4:**
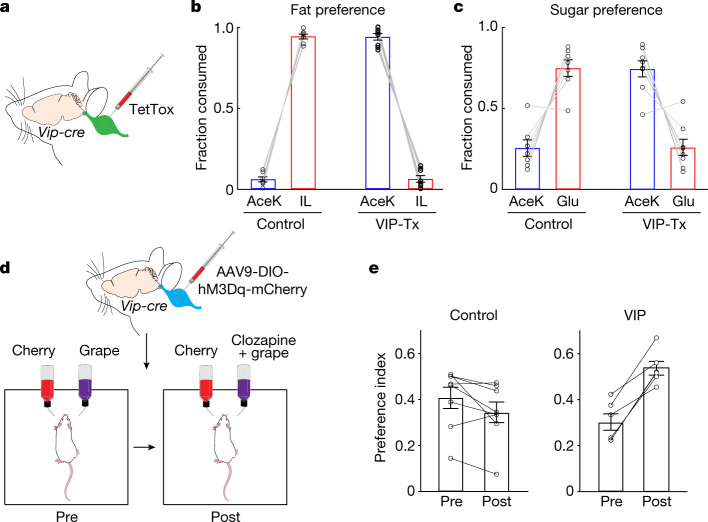
VIP vagal neurons convey sugar/nutrient preference. **a**, Silencing VIP neurons in the vagal ganglia by bilateral injection of AAV-DIO-TetTox into the nodose in *Vip-cre* mice. **b**,**c**, Fat and sugar preference tests for control mice and mice with silenced VIP-expressing vagal neurons (Vip-Tx). **b**, Control mice develop strong preference for fat during a standard 48 h fat-versus-sweetener test (*n* = 7). By contrast, silencing of VIP vagal neurons abolishes the development of fat preference (*n* = 8, Vip-Tx mice). Two-sided Mann–Whitney *U*-test, control versus Vip-Tx fat consumption, *P* = 3 × 10^−4^. **c**, Silencing of VIP vagal neurons also abolishes the development of sugar preference. Control (*n* = 7) versus silenced mice (*n* = 8). Two-sided Mann–Whitney *U*-test, control versus Vip-Tx sugar consumption, *P* = 6 × 10^−4^. Data are mean ± s.e.m. **d**, Strategy for chemogenetic activation of VIP vagal neurons. An excitatory DREADD receptor (via AAV-DIO-hM3Dq) was targeted bilaterally to the nodose of *Vip-cre* mice. The mice were then tested for their basal preference to cherry or grape flavour (pre). The mice were conditioned and retested using the less-preferred flavour plus the DREADD agonist clozapine (post) (Methods). **e**, Left, control mice (not expressing DREADD) presented with clozapine (5 mg l^−1^) in the less-preferred flavour do not switch their preference and maintain their basal, original flavour choice (*n* = 8 mice; two-tailed paired *t*-test, *P* = 0.061). Right, after associating clozapine-mediated activation of VIP vagal neurons with the less-preferred flavour, all the mice expressing DREADD switched their preference (*n* = 6 mice; two-tailed paired *t*-test, *P* = 9.6 × 10^−4^). Preference index values are mean ± s.e.m.

**Extended Data Fig. 8:**
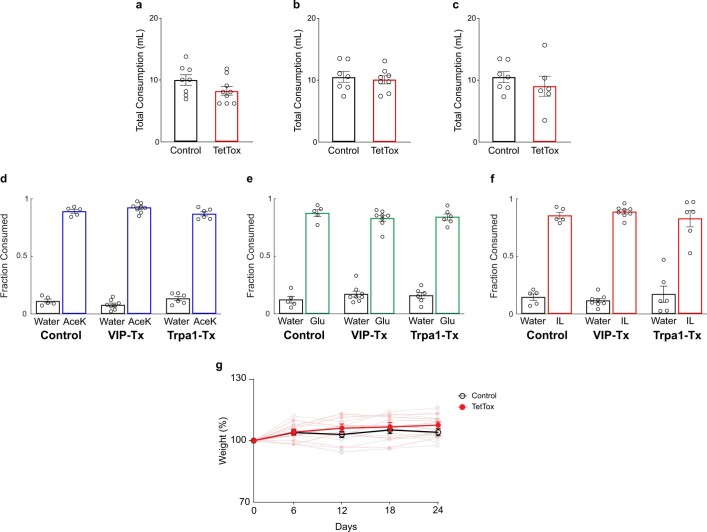
Drinking and eating in Tet-Tox silenced animals. **a**, Shown are graphs for consumption (AceK and IL) in two-bottle 48 h preference assay for control and cNST-silenced animals (*n* ≥ 8 mice), *P* = 0.151 (from Fig. 2a). **b**, Consumption in two-bottle 48 h preference assay for control and Vip-silenced mice (*n* ≥ 7 mice), *P* = 0.69 (from Fig. 4b). **c**, Consumption in two-bottle 48 h preference assay for control and Trpa1-silenced mice (*n* ≥ 6 mice), *P* = 0.44 (from Fig. 5c). Values are mean ± s.e.m. **d–f**, Animals with genetically silenced sugar/nutrient preference vagal neurons (VIP), or fat-only vagal neurons (Trpa1) still exhibit normal innate attraction to sweetener (**d**), sugar (**e**), and fat stimuli (**f**). Shown are graphs for 30 min two-bottle tests for control mice, and for mice with silenced VIP-expressing vagal neurons (VIP-Tx) and mice with silenced Trpa1-expressing vagal neurons (Trpa1-Tx). **d**, AceK versus water in VIP-Tx (*n* = 8) and Trpa1-Tx (*n* = 6) animals is not significantly different from controls. ANOVA with Tukey’s test: VIP-Tx, *P* = 0.36, Trpa1-Tx, *P* = 0.66. **e**, Glucose versus water in VIP-Tx (*n* = 8) and TrpA1-Tx (*n* = 6) is not significantly different from control animals. ANOVA with Tukey’s test: VIP-Tx, *P* = 0.45, Trpa1-Tx, *P* = 0.67. **f**, IL versus water in VIP-Tx (*n* = 8) and TrpA1-Tx (*n* = 6) is not significantly different from control animals. ANOVA with Tukey’s test: VIP-Tx, *P* = 0.87, Trpa1-Tx, *P* = 0.91. Values are mean ± s.e.m. Tastants: AceK (3 mM), Glucose (200 mM), IL (1.5%). **g**, The graph shows body weight measurements from *Vip-Cre* animals injected with AAV-Flex-TetTox in both nodose ganglia, from the time the animals were infected until the time behavioral preference tests were performed (days 24–26.); data is presented as percent change, with weight at time zero defined as 100%. Thin lines represent individual animals; dark lines represent the average body weight of TetTox (*n* = 7 mice, red) and control (*n* = 10 mice, black) animals. No significant differences were detected, two-way ANOVA, *P* = 0.37.

An important prediction is that inhibiting signalling from the nutrient-sensing vagal neurons should prevent the activation of the gut–brain axis, and consequently block the development of nutrient preference. Our strategy was to genetically silence the nutrient-sensing vagal neurons by bilaterally injecting the nodose of *Vip-cre*^38^ mice with an AAV-Flex-TetTox^4^ construct (Fig. 4a and Extended Data Fig. 3d). As hypothesized, blocking activity from these neurons markedly impaired the development of nutrient preference (Fig. 4b,c). Importantly, the immediate, innate attraction to sugar and fat in these mice was not affected (Extended Data Fig. 8).

Finally, we anticipated that artificial activation of this gut-to-brain nutrient preference circuit should afford the development of new preferences, in essence driving appetitive responses to previously unpreferred stimuli. To test this proposal, we bilaterally injected the nodose of *Vip-cre* mice with a Cre-dependent AAV virus encoding the excitatory designer receptor hM3Dq^39^, so that nutrient responding neurons could be experimentally activated by the DREADD agonist clozapine^40^. After allowing expression of DREADD (Extended Data Fig. 3e), mice were exposed to a preference assay using cherry- and grape-flavoured solutions (Fig. 4d), and to enhance attraction of these novel flavours, both solutions were spiked with an artificial sweetener (Methods). Next, we established a baseline preference for each mice (that is, grape vs cherry), introduced clozapine into the less-preferred flavour, and investigated whether clozapine-mediated activation of the nutrient-sensing neurons could create a new preference. Indeed, after 48 h of exposure to both solutions all of the mice markedly switched their preference to the clozapine containing flavour. By contrast, mice without the designer receptor did not develop a new preference, and if anything, were slightly averse to the DREADD activator (Fig. 4e). These results illustrate how non-natural activation of this gut–brain sugar/nutrient-sensing circuit can drive the development of a novel preference.

## Fat-only responders in the nodose

**Fig. 5:**
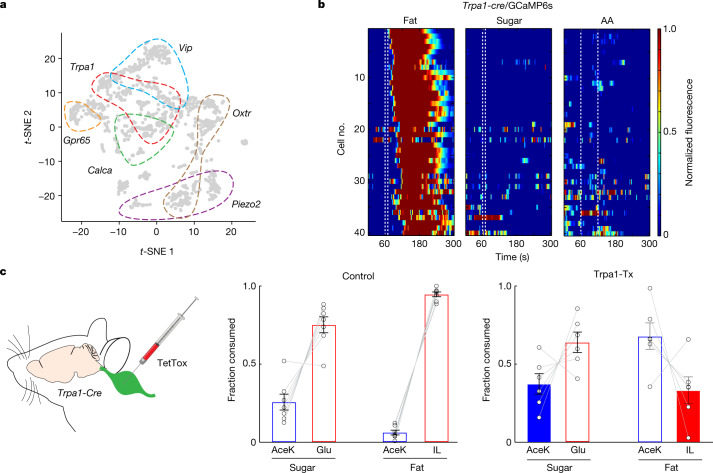
TRPA1 vagal neurons mediate fat-specific preference. **a**, Single-cell RNA-seq atlas of nodose ganglia^37^, showing vagal clusters for VIP (blue), *Trpa1* (red), *Gpr65 (*orange), *Calca* (green), *Oxtr* (brown) and *Piezo2* (purple). **b**, The vagal cluster expressing TRPA1 (Trpa1-GCaMP6s) responded selectively to intestinal delivery of fat (10% LA), but not sugar (500 mM Glu) or amino acid (250 mM amino acids mixture) stimuli. The heat maps show *z*-score-normalized fluorescence traces. Of 163 imaged neurons from 5 ganglia, approximately 24% responded to fat. See Extended Data Fig. 9 for imaging results for the other vagal clusters. **c**, Left, strategy for silencing of TRPA1 neurons in the vagal ganglia by bilateral injection of AAV-DIO-TetTox into the nodose of *Trpa1-cre* mice. Fat and sugar preference tests on control mice (middle) and mice with silenced TRPA1-expressing vagal neurons (Trpa1-Tx) (right). Control mice develop strong preference for fat and sugar after 48 h (*n* = 7). By contrast, silencing of TRPA1 vagal neurons abolishes the development of fat but not sugar preference (*n* = 6, right). Two-sided Mann–Whitney *U*-test, control versus Trpa1-Tx for sugar, *P* = 0.23; control versus Trpa1-Tx for fat, *P* = 1.1 × 10^−3^. Data are mean ± s.e.m.

**Extended Data Fig. 9:**
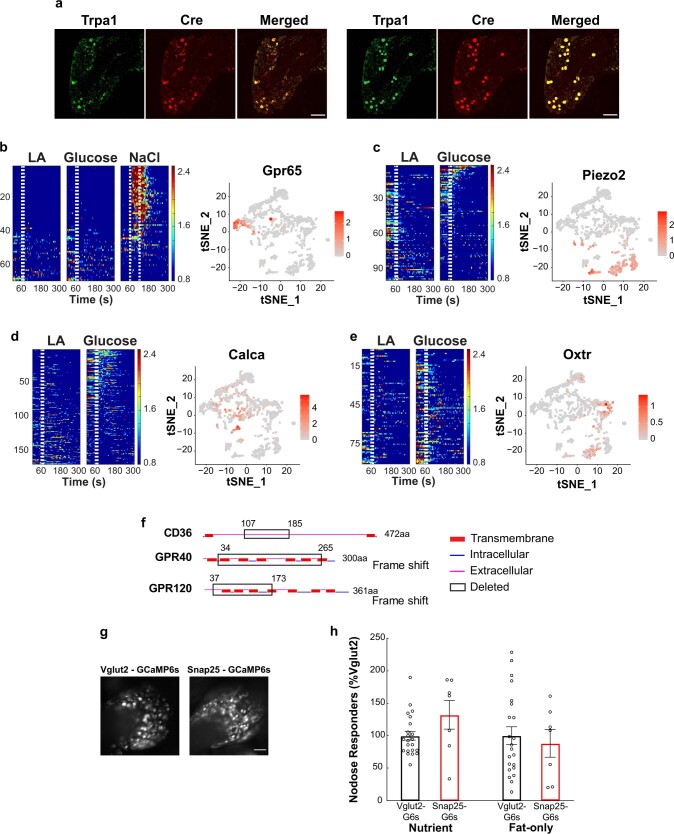
Gpr65, Piezo2, Calca, and Oxtr vagal neurons do not sense fat or sugar. **a**, Validation of *Trpa1-Cre* mice. Double *In situ* hybridization labeling for the endogenous Trpa1 gene (green) and for Cre-recombinase (red) in the nodose of *Trpa1-Cre* knock-in mice (see Methods). Shown is a frozen section demonstrating the strong overlap (*n* = 3 mice). The left 3 panels show the in-situ results, and the right 3 panels show an illustration of the labeling results. Scale bars, 100 μm. **b–e**, The panels show tSNE plots of the nodose transcriptome^37^ highlighting the 4 clusters, and heat maps of responses to intestinal delivery of fat and sugar from various vagal clusters using the corresponding Cre driver lines. **a**, GPR65 vagal neurons are known to indiscriminately respond to a wide range of long stimuli at high concentrations, including salt, fructose, mannose, and glucose, and considered osmolarity responders^4,22,58^. The heat maps show z-score-normalized fluorescence traces from all imaged vagal neurons in response to intestinal infusions of fat (10% linoleic acid, LA, 10 s), sugar (500 mM glucose, 10 s) or high osmolarity salt (1 M NaCl) for 60s in *Gpr65-Cre;Ai96* animals. Each row represents the average activity of a single cell to three trials. Stimulus window is shown by dotted white lines. *n* = 69 neurons from 3 ganglia. **c–e**, Calcium imaging of vagal responses in *Piezo2-Cre;Ai96*, *Calca-Cre;Ai96*, and *Oxtr-Cre;Ai96* animals. The heat maps showing z-score-normalized fluorescence traces of all imaged neurons in response to intestinal infusion of fat or sugar. **c**, Piezo2: *n* = 99 neurons from 4 ganglia; **d**, Calca: *n* = 168 neurons from 5 ganglia; **e**, Oxtr: *n* = 89 neurons from 6 ganglia. No significant responses were detected for any of the lines. **f**, Generation of fat receptor knockouts. Schematic illustrating the structural domains of the murine wild type CD36, GPR40, and GPR120 protein sequences, with the deletions denoted by the black boxes. For CD36 KO, we engineered a 626 nucleotide (nt) deletion removing residues 107 to 185, which forms part of the hydrophobic binding pocket of CD36^44^. For GPR40 KO, we engineered a 695 nt deletion that removed more than 75% of the protein. For GPR120, we introduced a 412 nt deletion removing 136 residues, and introducing a nonsense frameshift disrupting functional translation of the remaining two-thirds of the protein. See Methods for details. **g**, Representative views of GCaMP6s expressing neurons in vagal nodose imaging sessions using *Vglut2-Cre; Ai96* animals (left) or Snap25-GCaMP6s animals (right). Scale bar, 100 µm. Similar results were obtained from multiple animals. **h**, Comparisons of the fraction of sugar/nutrient responders (left) or fat-only responders (right), between *Vglut2-Cre; Ai96* (Vglut2-G6s, black, *n* = 22 ganglia) and Snap25-GCaMP6s animals (Snap25-G6s, red, *n* = 7 ganglia). No significant differences were found in vagal responses to intestinal delivery of fat or sugar between the Vglut2-G6s and Snap25-G6s genetic drivers (Two-sided Mann–Whitney U-test, *P* = 0.29 for sugar/nutrient responders, *P* = 0.83 for fat-only responders). All values are mean ± s.e.m.

We next investigated the identity of vagal neurons mediating the fat-only signals. Using the single-cell RNA-seq atlas from the nodose ganglion^35–37^, we searched for vagal neurons that did not express VIP (as the sugar-, fat- and amino acid-sensing marker), and identified five minimally overlapping candidate clusters (Fig. 5a): *Trpa1*, *Gpr65*, *Piezo2*, *Calca* and *Oxtr*. We engineered *Trpa1-cre* mice using the CRISPR–Cas9 system (Extended Data Fig. 9a and Methods), and obtained Cre driver lines for the other four candidates. Our results (Fig. 5b) demonstrated that the TRPA1-expressing vagal cluster responds selectively to intestinal delivery of fat, but not sugar or amino acid stimuli, thus defining the fat-only responders. Vagal neurons expressing GCaMP6s in *Gpr65-cre*, *Piezo2-cre*, *Calca-cre* or *Oxtr-cre* mice were unresponsive to intestinal delivery of sugar or fat stimuli (Extended Data Fig. 9b–e).

Next, we reasoned that genetic silencing of the fat-only circuit (that is, TRPA1-expressing vagal neurons) may abolish the development of fat preference but should have no effect on the development of sugar preference. Thus, we bilaterally injected the nodose of *Trpa1-cre* mice with an AAV-Flex-TetTox construct to silence the fat-only vagal neurons and tested the mice for sugar-versus-fat preference. Indeed, after genetic silencing, these mice no longer developed post-ingestive preference for fat stimuli, but retain their capacity to develop post-ingestive preference for sugar (Fig. 5c). Of note, their immediate attraction to fat was unaffected (Extended Data Fig. 8). Together, these results reveal the identity of the neurons mediating fat-only signals, and uncover their essential role in the gut-to-brain circuit mediating fat preference.

## Sugar and fat sensors in the gut

**Fig. 6:**
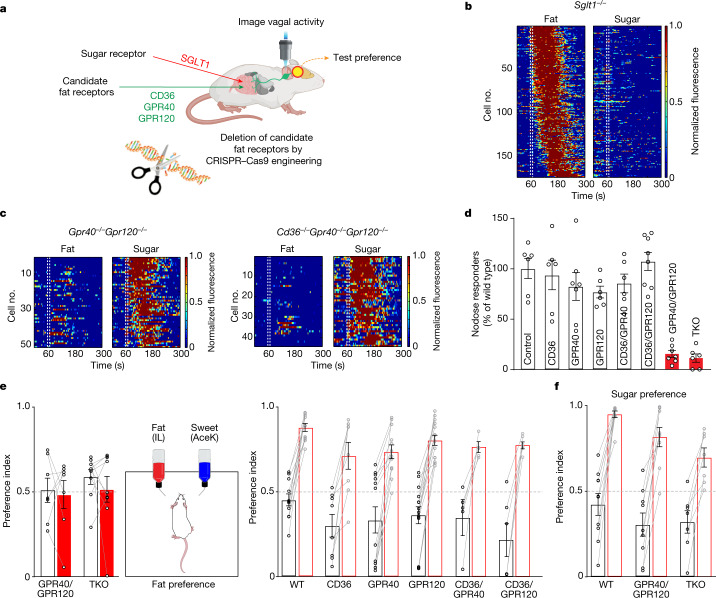
Intestinal GPR40 and GPR120 fat receptors activate the gut–brain axis. **a**, We engineered knockout mice for three candidate fat receptors in the gut, and generated mice with every combination of these knockouts. We then recorded vagal responses to intestinal delivery of fat (10% LA) and sugar (500 mM Glu), and tested them for the development of fat and sugar preference. **b**, Heat maps depict *z*-score-normalized fluorescence traces from vagal neurons of SGLT1-knockout mice in response to intestinal delivery of fat (10% LA) and sugar (500 mM Glu). As previously shown, SGLT1 functions as the gut-to-brain sugar receptor^4^, and no vagal neurons responded to sugar in the knockout mice. However, responses to fat were unaffected (*n* = 174 out of 903 imaged neurons from 10 ganglia). *Sglt1* is also known as *Slc5a1*. **c**, Heat maps illustrating the selective loss of fat responses in GPR40/GPR120 double-knockout (*n* = 51 out of 428 imaged neurons from 6 ganglia) and CD36/GPR40/GPR120 triple knockout (*n* = 44 out of 326 imaged neurons from 6 ganglia) mice. Note the normal responses to intestinal delivery of sugar in these knockout mice. See Extended Data Fig. 10 for imaging results for the other knockout lines. **d**, Bar graphs comparing vagal neurons responding to intestinal delivery of fat (10% LA) in control mice versus the various receptor knockouts (see Methods). Vagal responses were substantially affected only in the GPR40/GPR120 double-knockout (GPR40/GPR120, *n* = 7, *P* = 5 × 10^−6^) and in the triple knockout (TKO) (*n* = 6, *P* = 4 × 10^−6^) mice. Data are mean ± s.e.m.; statistics are shown in Methods. **e**, Knockout mice were tested for the development of fat preference. GPR40/GPR120 double knockouts (*n* = 7 mice, *P* = 0.81) and CD36/GPR40/GPR120 triple knockouts (*n* = 9 mice, *P* = 0.46) did not develop a preference for fat. White bars show initial preference and red bars show preference at the end of the 48 h test. All other combinations of knockouts developed a behavioural preference for fat, similar to control wild-type (WT) mice. Statistics are shown in Methods. Data are mean ± s.e.m. **f**, As expected, GPR40/GPR120 knockouts still develop preference for sugar. Wild type: *n* = 10 mice, *P* = 2.9 × 10^−5^; GPR40/GPR120: *n* = 9 mice, *P* = 8.0 × 10^−5^; TKO: *n* = 7 mice, *P* = 1.9 × 10^−3^. Data are mean ± s.e.m. Statistics are shown in Methods.

Pharmacological experiments have previously demonstrated that the sodium–glucose-linked transporter 1 (SGLT1) functions as the gut receptor that recognizes glucose and transmits the post-ingestive^41^, gut-to-brain sugar signals^4^. Here, we extend the specificity of these findings by generating SGLT1-knockout mice and examining their responses to intestinal stimulation with sugar and fat (Fig. 6a). The data shown in Fig. 6 demonstrate that all vagal responses to intestinal delivery of sugar are abolished in these mice. By contrast, the responses to fat stimuli remain unaffected.

We expected that the development of fat preference would depend on specific fat receptors expressed on the surface of intestinal EECs^42^. Dietary fat, once ingested and digested, is thought to be sensed by a number of putative gut receptors, including the fatty acid translocase CD36 (refs. ^43,44^) and the G protein-coupled receptors GPR40 (ref. ^45^) and GPR120 (refs. ^46,47^). We anticipated that one or more of these receptors would be used to transmit fat preference^46^ via the gut–brain axis. Therefore, we used CRISPR–Cas9 to generate mice deficient in all combinations of CD36, GPR40 and GPR120 (single, double and triple mutants) (Extended Data Fig. 9f; see Methods for details).

A key prediction would be that the loss of the essential receptor(s) would abolish vagal responses to intestinal stimulation with fat, thus defining the intestinal sensors for the gut-to-brain fat signals.

Because of the intricacies of breeding such a wide range of knockout combinations, and the need to introduce the GCaMP6s reporter for functional imaging into the various genetic backgrounds, we chose to use a direct fusion of GCaMP6s to *Snap25* regulatory sequences^48^ rather than crossing-in a Cre driver construct and a Cre-dependent GCaMP reporter. Our results showed that the Snap25-GCaMP6s construct is well expressed in vagal neurons, and compares favourably with our studies using other driver lines (Extended Data Fig. 9g,h).

**Extended Data Fig. 10:**
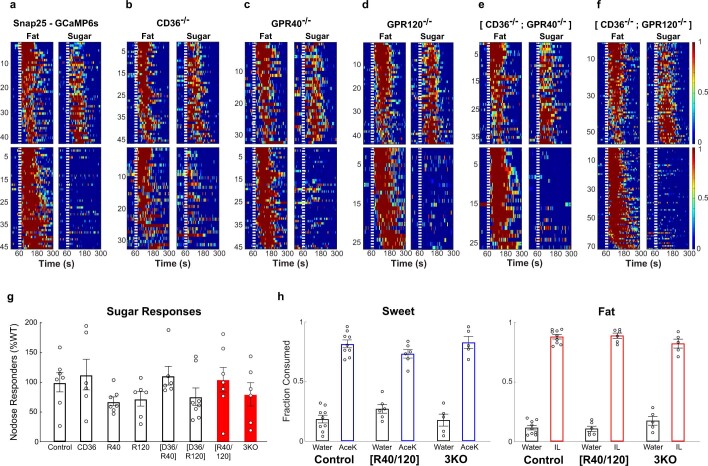
Functional imaging of vagal responses in fat receptor knockouts. **a-f**, Functional imaging of vagal neurons in response to intestinal delivery of fat (10% linoleic acid) and sugar (500 mM glucose) in Snap25-GCaMP6s mice harbouring various combinations of fat receptor deletions (see text for details). Heat maps show sugar/nutrient responders (top panels), and fat-only responders (bottom panels). **a**, control (*n* = 7 ganglia); **b**, CD36 KO (*n* = 6 ganglia); **c**, GPR40 KO (*n* = 7 ganglia); **d**, GPR120 KO (*n* = 6 ganglia); **e**, CD36 & GPR40 double KO (*n* = 6 ganglia); **f**, CD36 & GPR120 double KO (*n* = 8 ganglia). See Fig. 6c for GPR40 & GPR120 double KO and triple KO heat maps. **g**, Comparison of vagal responses to intestinal sugar stimuli in all fat receptor knockouts (see Fig. 6 for fat responses). ANOVA with Tukey’s HSD test to WT (*n* = 7): CD36 KO (*n* = 6 mice), *P* = 0.99; GPR40 KO (R40, *n* = 7 mice), *P* = 0.87; GPR120 KO (R120, *n* = 6 mice), *P* = 0.94; CD36/GPR40 double KO (CD36/R40, *n* = 6 mice), *P* = 0.99; CD36/GPR120 double KO, (CD36/R120, *n* = 8 mice), *P* = 0.96; GPR40/GPR120 double KO (R40/120, *n* = 7 mice), *P* = 0.99; CD36/GPR40/GPR120 triple KO (3KO, *n* = 6 mice), *P* = 0.99. Values are mean ± s.e.m. **h**, Fat receptor knockout animals that cannot transmit the gut-brain signal (GPR40/GPR120 double knockouts, and the triple knockout) still exhibit normal innate attraction to fat stimuli. Shown are brief-access (30 min) two-bottle tests for artificial sweetener (3 mM AceK) versus water (left panel), and fat (1.5% Intralipid, IL) versus water (right panel). ANOVA with Tukey’s test compared to wild type sweet consumption (*n* = 9): GPR40/GPR120 double KO (R40/R120): *n* = 6, *P* = 0.96; CD36/GPR40/GPR120 triple KO (D36/R40/R120): *n* = 5, *P* = 0.26. ANOVA with Tukey’s test compared to wild type fat consumption, R40/R120: *n* = 6, *P* = 0.25; *n* = 5, D36/R40/R120: *P* = 0.98. Two-tailed paired t-test. Values are mean ± s.e.m.

After testing all the fat receptor-deletion combinations (Fig. 6c,d and Extended Data Fig. 10a–f), we found that GPR40 and GPR120 were the essential mediators of intestinal fat signals to the vagal neurons. As expected, vagal neurons responding to sugar were unaffected in all of the mutants (Extended Data Fig. 10a–g). Notably, all fat responses—from both the fat-only and from the sugar-, fat- and amino acid-sensing vagal neurons—were abolished in the GPR40/GPR120 double-knockout mice, demonstrating that the same fat receptors are used in both gut-to-brain signalling pathways (that is, CCK-independent and CCK-dependent, respectively).

An expectation from these imaging results is that the GPR40/GPR120 double-knockout mice (as well as the triple-knockout mice) should not develop preference for fat^46^, whereas the various single mutants and the other double mutants should be unaffected. We note, however, that these are global knockouts, rather than conditional knockouts. Notably, GPR40, GPR120 and CD36 single mutants, as well as GPR40/CD36 and GPR120/CD36 double mutants were indistinguishable from control wild-type mice (Fig. 6e, right). By contrast, the GPR40/GPR120 double-knockout (and the triple-knockout) mice were no longer capable of developing a behavioural preference for fat (Fig. 6e, left). Importantly, the innate responses to fat stimuli were unaffected in the GPR40/GPR120 double and triple mutants, with the mice exhibiting a strong immediate attraction to fat, illustrating the fundamental difference between the taste and the gut–brain pathways (Extended Data Fig. 10h). As in control mice, fat receptor-knockout mice develop the normal preference for sugar^46^ (Fig. 6f). Together, these results demonstrate the function of GPR40 and GPR120 as the essential receptors signalling the presence of intestinal fat via the gut–brain axis.

## Discussion

Sugar and fat are indispensable nutrients, and it would be expected that dedicated circuits drive their consumption^1,4,13^. We have shown that in addition to the taste system, these nutrients rely on a dedicated gut-to-brain system to detect and report the presence of intestinal sugar and fat to the brain.

Here we demonstrate the fundamental role of these nutrient-sensing circuits by showing that genetic or pharmacological blockade of sugar and fat gut-to-brain signals, at any of the four stages following ingestion, abolished the development of nutrient preference: (1) by preventing sugar or fat binding to their corresponding intestinal receptors, (2) by blocking the activated gut cells from signalling to the vagal neurons, (3) by silencing the sugar- or fat-activated vagal neurons and preventing the transfer of their signals to the brain, and (4) by preventing the cNST neurons that receive the gut–brain signals from broadcasting the presence of intestinal sugar or fat to the rest of the brain.

An unexpected finding from these studies was the discovery of a single gut-to-brain pathway, based on CCK signalling, that functions as a generalist detector informing the brain of the intestinal presence of any of the three essential nutrients: sugar, fat and amino acids. Although each nutrient uses its own dedicated receptors in the gut, the convergence of the signal into a unique class of vagal neurons (VIP–UTS2b) highlights the simple and elegant logic of this circuit: after the gut cells are activated, the circuit does not need to preserve the identity of the specific nutrient stimulus, and needs only to ensure that the emerging gut–brain signal triggers behavioural preference^4^. Given that CCK functions as the signalling molecule in the gut for the sugar and nutrient-sensing pathway, we anticipate that there is a unique subset of intestinal CCK-positive EECs that co-express the sugar (SGLT1) and fat (GPR40 and GPR120) preference receptors (the nature of the amino acid receptor is not yet known). Notably, examination of single-cell RNA atlases from both rodent and human gut tissue suggests that this is probably the case^33,49^. Future studies should help to define this subtype of CCK-expressing EEC that uses CCK as a transmitter (rather than as a gut neuromodulator or hormone) to activate the gut–brain axis and report the presence of intestinal sugar, fat and amino acid nutrients.

Our results also uncovered two separate gut–brain circuits for intestinal fat sensing (that is, the fat-only and the sugar, fat and amino acid vagal pathways), yet both utilize the same receptors—GPR40 and GPR120—to drive the development of fat preference. Notably, silencing either circuit is sufficient to abolish the preference for fat, demonstrating that both are indispensable for the development of fat preference. Thus, activating the fat intestinal receptors only in the CCK-dependent pathway, or only in the CCK-independent (fat-only) pathway, is not sufficient on its own to trigger fat preference. Indeed, we measured cNST signals activated solely by the fat-only pathway, and they exhibited about 50% of the signal detected when both fat preference pathways were active (Extended Data Fig. 6g–i).

Given the essential role of sugar and fat in a healthy diet, and the importance of these gut–brain pathways in sugar and fat consumption (and most probably in over-consumption), it will be of great interest to determine the brain targets for each, and compare and contrast their function.

Finally, the identification of these gut receptors and gut–brain communication lines could help provide novel strategies to moderate the insatiable appetite for fat and sugar. Additionally, they clarify the fundamental difference between ‘liking’ and ‘wanting’^50^. Liking sweet and liking fat (that is, the innate attraction to these appetitive stimuli) is the result of activation of the taste system. Wanting sugar and fat, by contrast, is the gut–brain axis.

## Methods

### Animals

All procedures were carried out in accordance with the US National Institutes of Health (NIH) guidelines for the care and use of laboratory animals, and were approved by the Institutional Animal Care and Use Committee at Columbia University. Adult mice older than 6 weeks of age and from both sexes were used in all experiments. C57BL/6J (JAX 000664), TRAP2 (JAX 030323), *TRPM5* KO (JAX 013068), Ai96 (JAX 028866), Ai162 (JAX 031562), *Vglut2-IRES-cre* (JAX 028863), *Gpr65-IRES-cre* (JAX 029282), *Vip-IRES-cre* (JAX 010908); *Uts2b-cre* (JAX 035452); *Piezo2-cre* (JAX 027719); *Oxtr-cre* (JAX 031303); *Calca-cre* (JAX 033168); *Snap25-2A-GCaMP6s* (JAX 025111) and *Penk-IRES2-cre* (JAX 025112).

### Generation of genetically modified mice

To engineer *Trpa1-IRES-cre* knock-in mice^51^, a single guide RNA (sgRNA) (targeting CACAGAACTAAAAGTCCGGG) was selected to introduce an IRES-cre construct immediately downstream of the endogenous *Trpa1* stop codon. A single-stranded DNA donor containing gene-specific homology arms (150 bp each) and the IRES-cre fragment (Addgene #61574) was generated using the Guide-it Long ssDNA Production System (Takara Bio). Cas9 protein (100 ng μl^−1^), sgRNA (20 ng μl^−^^1^) and ssDNA donor (10 ng μl^−1^) were co-injected into the pronuclei of fertilized zygotes from B6CBAF1/J parents. Founder pups were screened for the presence of the knock-in allele using PCR, and candidates were validated by Sanger sequencing.

SGLT1-knockout mice were generated by co-injecting Cas9 mRNA (100 ng µl^−1^) with sgRNA (50 ng µl^−1^) targeting CGCATTGCGAATGCGCTCGT, resulting in a frameshift after the 20th residue and early termination after the 27th residue (wild-type SGLT1 is a 665-amino-acid protein). Homozygous SGLT1-knockout mice were bred and maintained on fructose-based rodent diet with no sucrose or cornstarch (Research Diets #D08040105). The mutant allele was validated by DNA sequencing.

To generate knockout mice for fat receptors (CD36, GPR40 and GPR120), Cas9 protein (50 ng µl^−1^) was co-injected with a total of 6 sgRNAs (7 ng µl^−1^ each: CD36: AAATATAACTCAGGACCCCG and TAGGATATGGAACCAAACTG; GPR40: AGTGAGTCGCAGTTTAGCGT and GAAGTTAGGACTCATCACAG; GPR120: CGACGCTCAACACCAACCGG and ACGCGGAACAAGATGCAGAG). The founder mice were validated by DNA sequencing and used to generate various homozygous knockout mice (that is, single, double and triple knockouts). All mutations in the individual homozygous lines were validated by DNA sequencing.

To engineer transgenic mice expressing Cre recombinase from the *Cckar* gene (*Cckar-cre* mice), a *cre* cassette was introduced at the ATG start codon of the *Cckar* gene using a 151 kb bacterial artificial chromosome (BAC) (RP23-50P5) carrying the *Cckar* gene, as described previously^52^.

### Fos stimulation and histology

Stimuli consisted of 20% Intralipid (sc215182, Santa Cruz Biotechnology), 10% linoleic acid, 10% oleic acid, 0.3% xanthan gum or 10% mineral oil. Stimuli were emulsified by dilution into milliQ water containing 0.1% xanthan gum and 0.05% Tween 80, and vortexed for a minimum of 10 min. Note that we used high concentration of Intralipid for Fos and TRAP2-labelling experiments to ensure enough Intralipid is consumed and digested during the 90 min stimulation window. By contrast, when performing 48 h behavioural tests examining the development of fat preference, a lower concentration of 1.5% Intralipid was used, particularly to ensure that the fat and the AceK (3 mM) are similarly attractive.

To motivate drinking behaviour during the 90 min Fos induction experiments, C57BL/6J mice were water-restricted for 23 h, given access to 1 ml of water for 1 h, and then water-restricted again for another 23 h. Previously, we showed that such water restriction prior to the 90 min drinking test did not affect the selectivity of cNST labelling^4^ (for example, no labelling in response to water or AceK; see also Extended Data Fig. 2). Mice had the full complement of food during water restriction (this is essential during Fos labelling experiments as food restriction would activate a wide range of additional circuits, including food-reward circuits upon presentation of sugar or fat stimuli). All Fos experiments consisted of 90 min of exposure to the stimuli; mice were housed individually and all the nesting material and food was removed from their cages. After 90 min, mice were perfused transcardially with PBS followed by 4% paraformaldehyde. Brains were dissected and fixed overnight in paraformaldehyde at 4 °C. The brains were sectioned coronally at 100 μm and labelled with anti-c-Fos (SYSY, no. 226004 guinea pig, 1: 5,000) diluted in 1× PBS with 5% normal donkey serum (EMD Millipore, Jackson ImmunoResearch) and 0.3% Triton X-100 for 48 h at 4 °C, and then Alexa Fluor 647-conjugated donkey anti-guinea pig (Jackson Immuno-Research) for 24 h at 4 °C. Images were acquired using an Olympus FluoView 1000 confocal microscope. Quantification of Fos labelling was carried out by recording the number of positive neurons in an equivalent 200 × 200 μm area of the cNST (bregma −7.5 mm) and area postrema.

For intragastric stimulation, the catheter was placed as previously described^4,53^. Mice were individually housed and allowed to recover for at least five days before stimulus delivery. A syringe pump micro-controller (Harvard Apparatus) was used to deliver 1.5 ml of the control PBS or 20% Intralipid solution^4^ at 0.050 ml min^−1^.

### Two-bottle preference assays

No behavioural experiments, including the short-term assays for taste responses, or the 48 h tests examining the development of sugar or fat preference used water-restricted or food-deprived mice. Mice were given ad libitum access to food and water for several days prior to the behavioural tests; any food or water restriction would severely affect the mice’s behaviour in preference or taste responses.

Development of fat preference: mice were first tested for their initial preference between 1.5% Intralipid and 3 mM AceK (pre testing) by completing 100 drinking trials. Each trial was initiated by the first lick and lasted for 5 s; the drinking ports then re-opened after 30 s of inter-trial interval. Next, mice were exposed to 500 licks to both 1.5% Intralipid and 3 mM AceK; this was repeated twice. Mice were then tested for the development of fat preference over 36 h using the 5 s trials. The pre- and post-preference indexes were calculated by dividing the number of licks to fat by the total lick count during the first 2–4 h (100 trials) of baseline measurements (pre) and during the last 2–4 h (100 trials) of the behavioural session (post), respectively.

In order to perform the two-bottle preference assay using large numbers of mice (for example, Figs. 4b, 5c and 6e), the setup was modified by using an LCD-based lick counter. The ‘pre’ preference index was calculated as the number of licks to fat divided by the total lick count during the first 4 h; the ‘post’ preference index was calculated as the number of licks to fat divided by the total lick count during the last 4 h of the session. Mice had ad libitum access to food throughout. The mice with a pre index >0.75 were not used owing to their high initial preference for fat (less than 20% of total tested mice had to be eliminated due to this strong bias).

### Fat, sugar and amino acid intestinal stimulation

Stimuli for nodose imaging experiments were as follows. Sugar: 500 mM glucose. Amino acids: a mix consisting of 50 mM methionine, 50 mM serine, 50 mM alanine, 50 mM glutamine and 50 mM cysteine dissolved in PBS. Fat: 10% linoleic acid, 10% linolenic acid, 10% hexanoic acid, 10% DHA, 10% oleic acid, diluted in PBS containing 0.1% xanthan gum and 0.05% Tween 80, and vortexed for a minimum of 10 min. Vehicle control: 0.1% xanthan gum and 0.05% Tween 80. For sugar and fat intestinal stimulation in imaging experiments, we used a 10 s window of stimulation; for amino acids, we used a 60 s stimulus, as we used lower concentrations of each in the mix of several amino acids (see above).

Note that if using Intralipid mix^12,13^ (a 20% soybean oil emulsion, Santa Cruz) for nodose imaging experiments (rather than consumption where it would be naturally digested and broken down into short, medium and long chain fatty acids), the material needed to be pre-digested with lipases (mimicking its natural course of action upon ingestion). Using undigested complex oils for intestinal stimulation in imaging experiments yielded inaccurate and unreliable responses (data not shown). Intralipid was incubated with 4 mg ml^−1^ lipase (sigma) in PBS plus 10 mM CaCl_2_ for a minimum of 5 h at 37 °C.

### Stereotaxic surgery

Mice were anaesthetized with ketamine and xylazine (100 mg kg^−1^ and 10 mg kg^−1^, intraperitoneal), and placed into a stereotaxic frame with a closed-loop heating system to maintain body temperature. The coordinates (Paxinos stereotaxic coordinates) used to inject and place recording fibres in the cNST were: caudal 7.5 mm, lateral ±0.3 mm, ventral 3.7–4 mm, all relative to Bregma. The fibre photometry experiments used a 400 μm core, 0.48 NA optical fibre (Doric Lenses) implanted 50–100 μm over the left cNST. TRAP2 mice were stereotaxically injected bilaterally in the cNST with AAV9-Syn-DIO-mCherry (300 nl per mouse), AAV9 DIO eGFP-RPL10a (300 nl per mouse) or AAV9 CBA.FLEX-TetTox^54^ (300 nl per mouse).

### Genetic access to fat preference neurons in the brain

The TRAP strategy was used in TRAP2^20,55^ mice to gain genetic access to fat-activated neurons in the cNST. A minimum of 5 days after injection, the AAV-injected TRAP2 mice or TRAP2; Ai9 mice were water-restricted for 23 h, given access to 1 ml of water for 1 h, water-restricted again for another 23 h (with ad libitum food), and then presented with 20% Intralipid ad libitum in the absence of food and nesting material. After 1 h, mice were injected intraperitoneally with 12.5 mg kg^−1^ 4-hydroxytamoxifen (Sigma H6278) and placed back in the same cage for an additional 3 h. Following 4 h of Intralipid exposure, mice were returned to regular home-cage conditions (group-caged, with nesting material, ad libitum food and water). Mice were used for experiments a minimum of 10 days after this TRAP protocol. C57BL/6J and TRAP2 mice expressing TetTox in the cNST were tested in the two-bottle Intralipid versus sweetener preference assay for 48 h, as described previously^4^. Note that mice were never food-deprived prior to TRAPping, so as to prevent unrelated labelling and confounds from the activation of feeding and food-reward responding neurons.

### Fibre photometry

*Vglut2-cre;Ai96* mice were placed in a stereotaxic frame and implanted with a 400 μm core, 0.48 NA optical fibre (Doric Lenses) 50–100 μm over the left cNST. Photometry experiments were conducted as described previously^4,56^. To quantify the effects of vagotomy, we calculated the ratio of stimulus-related peak amplitude of the normalized trace (within 120 s of stimulus onset) prior vagotomy versus after vagotomy.

The duodenal catheterization surgery was carried out as described previously^4^. Stimulus delivery was performed via a series of peristaltic pumps (BioChem Fluidics) operated via custom Matlab software and Arduino microcontroller. Stimuli and washes were delivered through separate lines that converged on a common perfusion manifold (Warner Instruments) connected to the duodenal catheter. Trials consisted of a 60-s baseline (PBS 200 μl min^−1^), a 30 s stimulus (200 μl min^−1^), and a 3-min washout period (150 s at 600 μl min^−1^, and 30 s at 150 μl min^−1^). Stimuli were each presented three times in an interleaved fashion. The vagotomy procedure was carried out after the first round of stimulus as described previously^4,57^.

### Nodose ganglion injection experiments

#### Genetic vagal silencing experiments

Cre-expressing mice (*Vip-cre* and *Trpa1-cre*) were anaesthetized with ketamine and xylazine (100 mg kg^−1^ and 10 mg kg^−1^, intraperitoneal). The skin under the neck was shaved and betadine and alcohol were used to scrub the skin three times. A midline incision (~1.5 cm) was made and the trachea and surrounding muscles were gently retracted to expose the nodose ganglia. AAV9 CBA.FLEX-TetTox (600 nl per ganglion) containing Fast Green (Sigma, F7252-5G) was injected in both left and right ganglia using a 30° bevelled glass pipette (custom-bevelled Clunbury Scientific). At the end of surgery, the skin incision was closed using 5-0 absorbable sutures (CP medical, 421A). Mice were allowed to recover for a minimum of 26 days before 2-bottle preference tests for sugar and fat. We note that almost all of the *Vip-cre* mice survived the surgical procedure and bilateral injections, whereas only 50% of the *Trpa1-cre* mice survived.

The *Trpa1-cre* knock-in line was validated by in situ hybridization experiments (Extended Data Fig. 9a). Fixed frozen nodose ganglia were sectioned at 16 μm thickness and processed for mRNA detection using the RNAscope Fluorescent Multiplex Kit (Advanced Cell Diagnostics) following the manufacturer’s instructions. The following RNAscope probes were used: Trpa1 (catalogue no. 400211-C3) and Cre-O4 (catalogue no. 546951).

#### Chemogenetic activation experiments

For gain-of-preference experiments, *Vip-cre* mice were injected bilaterally with 600 nl per ganglion of an AAV carrying the Cre-dependent activator DREADD (AAV9-Syn-DIO-hM3Dq-mCherry)^37,39^ and were allowed to recover for a minimum of three weeks before behavioural tests. Control and *Vip-cre* mice were tested in a two-bottle grape versus cherry flavour-preference assay (grape: 0.39 g l^−1^ Kool-Aid Unsweetened Grape, cherry: 0.36 g l^−1^ Kool-Aid Unsweetened Cherry, both containing 1 mM AceK). Flavour-preference tests were carried out as previously described^4^.

### Vagal calcium imaging

Each mouse was anaesthetized with ketamine (100 mg kg^−1^) and xylazine (10 mg kg^−1^). The mice were tracheotomized, and the nodose ganglion was exposed for imaging exactly as previously described^4^.

For CCKAR blocker experiments, devazepide (Sigma) was dissolved in DMSO and diluted to a final dose of 4 mg kg^−1^ in saline^11^. For glutamate receptor blocker experiments, a mixture of metabotropic glutamate receptor antagonist AP3 (2 mg kg^−1^) and ionotropic glutamate receptor antagonist kynurenic acid (300 μg kg^−1^) was used. CCKAR and glutamate receptor blockers were delivered both into the intestines and abdominal cavity^11^; after a 5 min incubation period, the imaging session was started. For CCK application, the intestines, still attached to the anaesthesized mouse, were partly placed on a 25 mm petri dish to allow delivery (60 s) and washout (>180 s) of the stimuli (1 μg ml^−1^ CCK peptide; Bachem 4033101).

Note that for nodose imaging experiments using sugar, glucose stimuli consisted of 10 s pulses since stimulating with high concentration (>250 mM) for long pulses (60 s or more) strongly activates nutrient-independent vagal responses^4,22,58^, severely masking sugar/nutrient-evoked responses.

### Calcium imaging data collection and analysis

Imaging data was obtained using an Evolve 512 EMCCD camera (Photo-metrics). Data was acquired at 5 Hz. A single field of view was chosen for recording and analysis from each ganglion. Calcium imaging data collected at 5 Hz was downsampled by a factor of 2, and the images were stabilized using the NoRMCorre algorithm^59^. Motion-corrected movies were then manually segmented in ImageJ using the Cell Magic Wand plugin. Neuropil fluorescence was subtracted from each region of interest with the FISSA toolbox^60^, and neural activity was denoised using the OASIS deconvolution algorithm^61^.

Neuronal activity was analysed for significant stimulus-evoked responses as described previously^4,62^. Note that for the fat receptor-knockout imaging studies, the minimal peak amplitude for defining responders was set to 1% Δ*F*/*F*. To quantify responses in fat receptors knockouts (Fig. 6d and Extended Data Fig. 10g), the number of responding neurons over the total number of imaged neurons per ganglia was normalized to the number of responders in wild-type control mice (set to 100%).

For experiments using blockers, two repeat trials per stimuli were used to accommodate the expanded time scale of the session (that is, before and after), and a neuron was considered a responder if it responded in both trials. The two-trial average area under curve for each stimulus was used to quantify the before and after responses (Extended Data Fig. 6d,e).

Imaging data is presented as heat maps of *z*-score-normalized responses (see also ref. ^4^). Equivalent results are obtained when using absolute Δ*F*/*F* (data not shown)

### Statistics

No statistical methods were used to predetermine sample size, and investigators were not blinded to group allocation. No method of randomization was used to determine how mice were allocated to experimental groups. Statistical methods used include one-way ANOVA followed by Tukey’s honest significant difference post hoc test, two-tailed *t*-test, two-way ANOVA or the two-sided Mann–Whitney *U*-test, and are indicated for all figures. Analyses were performed in MATLAB and GraphPad Prism 8. Data are presented as mean ± s.e.m.

Figure 6d: ANOVA with Tukey’s test compared to Snap25-GCaMP6s control. CD36 KO (*n* = 6 mice) vs control, *P* = 0.99; GPR40 KO (*n* = 7 mice) vs control, *P* = 0.89; GPR120 KO (*n* = 6 mice) vs control, *P* = 0.53; CD36/GPR40 double KO (*n* = 6 mice) vs control, *P* = 0.96; CD36/GPR120 double KO, (*n* = 8 mice) vs control, *P* = 0.99; GPR40/GPR120 double KO (*n* = 7 mice) vs control, *P* = 5 × 10^−6^; CD36/GPR40/GPR120 triple KO (*n* = 6 mice) vs control, *P* = 4 × 10^−6^.

Figure 6e: Two-tailed paired *t*-tests evaluating pre versus post fat preference. Wild-type mice (*n* = 11 mice) pre vs post, *P* = 2 × 10^−6^; CD36 KO (*n* = 8 mice) pre vs post, *P* = 4.8 × 10^−3^; GPR40 KO (*n* = 12 mice) pre vs post, *P* = 1 × 10^−4^; GPR120 KO (*n* = 14 mice) pre vs post, *P* = 1.03 × 10^−4^; CD36/GPR40 KO (*n* = 5 mice) pre vs post, *P* = 2 × 10^−2^; CD36/GPR120 KO (*n* = 6 mice) pre vs post, *P* = 1.7 × 10^−3^; GPR40/GPR120 double KO (*n* = 7 mice) pre vs post, *P* = 0.81; CD36/GPR40/GPR120 triple KO (*n* = 9 mice) pre vs post, P = 0.46.

Figure 6f: Two-tailed paired *t*-tests evaluating pre versus post sugar preference. Wild-type mice (*n* = 10 mice) pre vs post, *P* *=* 2.9 × 10^−5^; *Gpr40*^*−/−*^*Gpr120*^*−/−*^ (*n* *=* 9 mice), pre vs post, *P* *=* 8.0 × 10^−5^; *Cd36*^*−/−*^*Gpr40*^*−/−*^*Gpr120*^*−*^^*/−*^ (*n* *=* 7 mice), pre vs post, *P* *=* 1.9 × 10^−3^.

### Reporting summary

Further information on research design is available in the Nature Research Reporting Summary linked to this article.

## Online content

Any methods, additional references, Nature Research reporting summaries, source data, extended data, supplementary information, acknowledgements, peer review information; details of author contributions and competing interests; and statements of data and code availability are available at 10.1038/s41586-022-05266-z.

## Supplementary information

Reporting Summary

## Data Availability

All data supporting the findings of this study are available upon request.

## Code availability

Custom code is available from corresponding author upon request.
