# Supplementary material for: Gut–brain circuits for fat preference
Source: Nature. 2022 Sep 7;610(7933):722–30. doi: 10.1038/s41586-022-05266-z (PMC9605869; doi:10.1038/s41586-022-05266-z)
Supplement: Supplementary file 1 — Reporting Summary [file 41586_2022_5266_MOESM1_ESM.pdf]

nature portfolio

Corresponding author(s): Charles S Zuker

Last updated by author(s): Aug 6, 2022

Reporting Summary

Nature Portfolio wishes to improve the reproducibility of the work that we publish. This form provides structure for consistency and transparency in reporting. For further information on Nature Portfolio policies, see our Editorial Policies and the Editorial Policy Checklist.

Statistics

For all statistical analyses, confirm that the following items are present in the figure legend, table legend, main text, or Methods section.

n/a	Confirmed	
☐	☒	The exact sample size (*n*) for each experimental group/condition, given as a discrete number and unit of measurement
☐	☒	A statement on whether measurements were taken from distinct samples or whether the same sample was measured repeatedly
☐	☒	The statistical test(s) used AND whether they are one- or two-sided<br>*Only common tests should be described solely by name; describe more complex techniques in the Methods section.*
☒	☐	A description of all covariates tested
☒	☐	A description of any assumptions or corrections, such as tests of normality and adjustment for multiple comparisons
☐	☒	A full description of the statistical parameters including central tendency (e.g. means) or other basic estimates (e.g. regression coefficient) AND variation (e.g. standard deviation) or associated estimates of uncertainty (e.g. confidence intervals)
☐	☒	For null hypothesis testing, the test statistic (e.g. *F*, *t*, *r*) with confidence intervals, effect sizes, degrees of freedom and *P* value noted<br>*Give P values as exact values whenever suitable.*
☒	☐	For Bayesian analysis, information on the choice of priors and Markov chain Monte Carlo settings
☒	☐	For hierarchical and complex designs, identification of the appropriate level for tests and full reporting of outcomes
☒	☐	Estimates of effect sizes (e.g. Cohen's *d*, Pearson's *r*), indicating how they were calculated

Our web collection on statistics for biologists contains articles on many of the points above.

Software and code

Policy information about availability of computer code

Data collection	Tucker-Davis Technologies Synapse (Version 90-39473P), MicroManager (Version 1.4), Olympus Fluoview (FV10), Arduino IDE (Version 1.8.15), MathWorks Matlab (R2019a, R2019b)
Data analysis	MathWorks Matlab (R2019a, R2019b), FIJI (Version 1.53c), GraphPad Prism 8.4.3

For manuscripts utilizing custom algorithms or software that are central to the research but not yet described in published literature, software must be made available to editors and reviewers. We strongly encourage code deposition in a community repository (e.g. GitHub). See the Nature Portfolio guidelines for submitting code & software for further information.

Data

Policy information about availability of data

All manuscripts must include a data availability statement. This statement should provide the following information, where applicable:

- Accession codes, unique identifiers, or web links for publicly available datasets
- A description of any restrictions on data availability
- For clinical datasets or third party data, please ensure that the statement adheres to our policy

All data supporting the findings of this study are available upon request.

Human research participants

Policy information about studies involving human research participants and Sex and Gender in Research.

Reporting on sex and gender	*Use the terms sex (biological attribute) and gender (shaped by social and cultural circumstances) carefully in order to avoid confusing both terms. Indicate if findings apply to only one sex or gender; describe whether sex and gender were considered in study design whether sex and/or gender was determined based on self-reporting or assigned and methods used. Provide in the source data disaggregated sex and gender data where this information has been collected, and consent has been obtained for sharing of individual-level data; provide overall numbers in this Reporting Summary. Please state if this information has not been collected. Report sex- and gender-based analyses where performed, justify reasons for lack of sex- and gender-based analysis.*
Population characteristics	*Describe the covariate-relevant population characteristics of the human research participants (e.g. age, genotypic information, past and current diagnosis and treatment categories). If you filled out the behavioural & social sciences study design questions and have nothing to add here, write "See above."*
Recruitment	*Describe how participants were recruited. Outline any potential self-selection bias or other biases that may be present and how these are likely to impact results.*
Ethics oversight	*Identify the organization(s) that approved the study protocol.*

Note that full information on the approval of the study protocol must also be provided in the manuscript.

Field-specific reporting

Please select the one below that is the best fit for your research. If you are not sure, read the appropriate sections before making your selection.

☒ Life sciences ☐ Behavioural & social sciences ☐ Ecological, evolutionary & environmental sciences

For a reference copy of the document with all sections, see nature.com/documents/nr-reporting-summary-flat.pdf

Life sciences study design

All studies must disclose on these points even when the disclosure is negative.

Sample size	Sample size was determined based similar studies in the literature and our experience. No statistical method was used to determine the sample size prior to the study.
Data exclusions	Animals in which post-hoc histological examination showed that viral targeting or the position of implanted fiber were in the incorrect location were excluded from analysis. This exclusion criteria was predetermined.
Replication	We performed multiple independent experiments as noted in the figure legends. Results were reproducible.
Randomization	Stimuli order was random, otherwise in situations as described in the manuscript where no randomization was used, the stimuli were interspersed and repeated among trials.
Blinding	Investigators were not blinded to group allocation, as data analysis was performed automatically with the same scripts executed for each experimental group.

Reporting for specific materials, systems and methods

We require information from authors about some types of materials, experimental systems and methods used in many studies. Here, indicate whether each material, system or method listed is relevant to your study. If you are not sure if a list item applies to your research, read the appropriate section before selecting a response.

Materials & experimental systems

n/a	Involved in the study
☐	☒ Antibodies
☒	☐ Eukaryotic cell lines
☒	☐ Palaeontology and archaeology
☐	☒ Animals and other organisms
☒	☐ Clinical data
☒	☐ Dual use research of concern

Methods

n/a	Involved in the study
☒	☐ ChIP-seq
☒	☐ Flow cytometry
☒	☐ MRI-based neuroimaging

Antibodies

Antibodies used	anti c-Fos (Synaptic Systems, 226004, Guinea Pig, 1:5000),
Validation	antibodies has been validated extensively, e.g. by immuno-staining on mouse brain sections (Song, et al. Science advances, 52: eaat 3210, (2019)).

Animals and other research organisms

Policy information about studies involving animals; ARRIVE guidelines recommended for reporting animal research, and Sex and Gender in Research

Laboratory animals	Adult animals 6-24 weeks of age and from both genders were used in experiments. C57BL/6J (JAX 000664), TRAP2 (JAX 030323), TRPM5 KO (JAX 013068), Ai96 (JAX 028866), Ai162 (JAX 031562), VGlut2-IRES-Cre (JAX 028863), Gpr65-IRES-Cre (JAX 029282), Vip-IRES-Cre (JAX 010908); Uts2b-Cre (JAX 035452); Piezo2-Cre (JAX 027719); Oxtr-Cre (JAX 031303); Calca-Cre (JAX 033168); Snap25-2A-GCaMP6s (JAX 025111), Penk-IRES2-Cre (JAX 025112).
Wild animals	No wild animals were used.
Reporting on sex	Animals of both sexes were used in the behavioral and imaging studies, without bias.
Field-collected samples	No field-collected samples were used.
Ethics oversight	All procedures were carried out in accordance with the US National Institutes of Health (NIH) guidelines for the care and use of laboratory animals, and were approved by the Institutional Animal Care and Use Committee at Columbia University.

Note that full information on the approval of the study protocol must also be provided in the manuscript.
